# Structural Characterization of Alumina-Supported Rh Catalysts: Effects of Ceriation and Zirconiation by using Metal–Organic Precursors

**DOI:** 10.1002/cphc.201300537

**Published:** 2013-08-13

**Authors:** Anna B Kroner, Mark A Newton, Moniek Tromp, Andrea E Russell, Andrew J Dent, John Evans

**Affiliations:** a Diamond Light Source, Chilton,Oxfordshire, OX11 0DE (UK); b The European Synchrotron Radiation Facility,Grenoble, 38043 (France); c Technische Universitat Munchen,Lichtenbergstrasse 4, 85748 Garching (Germany); d School of Chemistry, University of Southampton,Southampton, SO17 1BJ (UK); e Research Complex at Harwell, Rutherford Appleton Laboratory,Didcot, OX11 1FA (UK)

**Keywords:** cerium, EXAFS spectroscopy, photoelectron spectroscopy, rhodium, zirconium

## Abstract

The effects of the addition of ceria and zirconia on the structural properties of supported rhodium catalysts (1.6 and 4 wt % Rh/γ-Al_2_O_3_) are studied. Ceria and zirconia are deposited by using two preparation methods. Method I involves the deposition of ceria on γ-Al_2_O_3_ from Ce(acac)_3_, and the rhodium metal is subsequently added, whereas method II is based on a controlled surface reaction technique, that is, the decomposition of metal–organic M(acac)_*x*_ (in which M=Ce, *x*=3 and M=Zr, *x*=4) on Rh/γ-Al_2_O_3_. The structures of the prepared catalyst materials are characterized ex situ by using N_2_ physisorption, transmission electron microscopy, high-angle annular dark-field scanning transmission election microscopy, energy-dispersive X-ray spectroscopy, X-ray photoelectron spectroscopy (XPS), and X-ray absorption fine structure spectroscopy (XAFS). All supported rhodium systems readily oxidize in air at room temperature. By using ceriated and zirconiated precursors, a larger rhodium-based metallic core fraction is obtained in comparison to the undoped rhodium catalysts, suggesting that ceria and zirconia protect the rhodium particles against extensive oxidation. XPS results indicate that after the calcination and reduction treatments, a small amount of chlorine is retained on the support of all rhodium catalysts. EXAFS analysis shows significant Rh—Cl interactions for Rh/Al_2_O_3_ and Rh/CeO_*x*_/Al_2_O_3_ (method I) catalysts. After reaction with H_2_/He in situ, for series of samples with 1.6 wt % Rh, the EXAFS first shell analysis affords a mean size of approximately 30 atoms. A broader spread is evident with a 4 wt % rhodium loading (ca. 30–110 atoms), with the incorporation of zirconium providing the largest particle sizes.

## 1. Introduction

Rhodium has long been implemented as a core component in the so-called three-way automotive exhaust catalyst (TWC) as a result of its excellent thermal stability, poison resistance, and superior selectivity for NO_*x*_ removal.^1^–^3^ A wide range of rhodium compounds, for example, single crystal and polycrystalline rhodium surfaces^4^–^8^ as well as supported rhodium particles,^9^–^12^ have been used to build reactivity models of highly dispersed systems. Studies over single crystals have been performed with a high degree of control over the surface and molecular/kinetic specificity. However, the behaviour of supported rhodium catalysts has been found to present more complex structures than that of the rhodium single crystal surfaces under equivalent conditions;^13^–^15^ therefore, further study of the structure and catalytic reactivity of dispersed catalysts is required.

Owing to the high activity and selectivity of supported rhodium catalysts, these materials are widely used for reactions such as the hydrogenation of CO, the reduction of NO_*x*_ to N_2_, the CO–NO reaction, and the water gas shift reaction (WGSR).^11^, ^16^, ^17^ The overall catalytic performance and catalyst lifetime are significantly improved by doping the catalyst with ceria and/or zirconia. CeO_2_-supported noble-metal catalysts are capable of storing oxygen under oxidizing conditions and releasing oxygen under reducing conditions through the facile conversion between the Ce^4+^ and Ce^3+.[18]^ This feature is strongly related to the creation, healing, and diffusion of oxygen vacancies, especially at the ceria surfaces.^19^ The repeated redox (Lambda) cycling, which TWCs endure under working conditions,^20^ places the Rh–Ce interface under significant stress because of the continuous changes in the lattice parameters (owing to the larger Ce^3+^ ionic radius compared to that of Ce^4+^), which can easily induce the formation of structural defects, thereby promoting oxygen mobility in the framework.^21^

Doping with ceria–zirconia, rather than with pure CeO_2_, is now widely used in TWCs as it produces a superior thermal stability and oxygen-storage capacity (OSC).^22^–^24^ Balducci et al.^25^ reported that the introduction of Zr into a CeO_2_ lattice lowers the energy for Ce^4+^ reduction and leads to easier diffusion of oxygen from the bulk to the surface, thereby promoting the redox action of the Ce^4+^/Ce^3+^ couple. The partial OSC has been found to increase with increasing amounts of dissolved ZrO_2_ in CeO_2_.^23^ One explanation for the increased OSC of the mixed oxide involves a geometric effect, wherein the smaller radius of Zr^4+^ favors the presence of Ce^3+^ ions, eliminating the strain associated with their formation.^25^, ^26^ The addition of ZrO_2_ increases the thermal stability of CeO_*x*_ during catalytic processes, which easily sinters above 1073 K, particularly under reducing conditions.^27^, ^28^

The requirements of high selectivity and activity, which TWCs must fulfill, are amongst the most crucial demands for a successful commercial application. Thus, it is essential to study the interaction between the active metal, for example Rh, and the different promoters, such as Ce and Zr, in order to understand the relevant physical and chemical mechanisms of the different catalyst components and how they influence each other during their catalytic action in a TWC.

Rhodium nanoparticle systems on γ-alumina, promoted by ceria and/or zirconia, are predominantly amorphous or poorly crystalline materials. In this study, structural characterization of the supported rhodium catalysts is performed by using N_2_ physisorption, transmission electron microscopy (TEM), high-angle annular dark-field scanning transmission election microscopy (STEM–HAADF), X-ray photoelectron spectroscopy (XPS), X-ray absorption near-edge structure (XANES) studies, and extended X-ray absorption fine structure spectroscopy (EXAFS). Two doping methods are used: method I involves ceriation of the alumina support by using Ce-based precursors, followed by deposition of Rh. Initially, a series of γ-Al_2_O_3_/CeO_*x*_ supports are prepared by using three different Ce precursors: (NH_4_)_2_Ce(NO_3_)_6_, Ce(NO_3_)_3_, and Ce(acac)_3_. The scanning electron microscopy energy-dispersive X-ray spectroscopy (SEM–EDX) analyses shows that supports produced from the metal–organic precursor Ce(acac)_3_ provides an apparently uniform coverage of the γ-alumina particles, unlike our observations with inorganic precursors. Therefore, only the Ce(acac)_3_ precursor is pursued further in this study. Method II is based on the surface organometallic chemistry of metals to promote local decomposition of the additives. CeO_*x*_ is deposited on a Rh/Al_2_O_3_ catalyst that has previously been synthesized through incipient wetness impregnation.^29^ In this reverse ordering, the rhodium particles may act as seed points for the decomposition of the Ce complex,^29^ locating the Ce directly onto, or in close proximity to, the active rhodium metal. Method II can also be extended to prepare the zirconiated catalyst Rh/ZrO_2_/γ-Al_2_O_3_ and the doubly promoted Rh/CeO_*x*_/ZrO_2_/γ-Al_2_O_3_ catalyst by using Zr(acac)_4_ as the precursor.

## 2. Results

### 2.1 Surface Area Measurements

The Brunauer–Emmett–Teller (BET) surface area of the supports and catalysts were measured by using N_2_ adsorption; the results are presented in Table S1 of the Supporting Information, together with an example isotherm (Figure S1). The BET surface areas measured for γ-alumina and ceria- and/or zirconia-doped γ-Al_2_O_3_ were approximately 90 m^2^ g^−1^, which is in good agreement with the literature results (96 m^2^ g^−1^ for 0.5 wt % Rh/γ-Al_2_O_3_) reported by McCabe et al.^30^ The BET surface areas of the ceriated rhodium catalysts shows that Rh/CeO_*x*_/Al_2_O_3_ (method I) results in a lower total surface area (68–70 m^2^ g^−1^) than Rh/CeO_*x*_/Al_2_O_3_ (method II) and undoped rhodium on alumina (82–91 m^2^ g^−1^). The reduced BET surface areas for the samples obtained by using method I can be attributed to γ-Al_2_O_3_ particle sintering induced by the 773 K calcinations that were used. The adsorption/desorption isotherms for the rhodium catalysts indicate that there is no mesoporosity present in the structure of any of the samples. The surface area largely originates from the external surface area of the very fine particles, and there is little microporosity in the type of alumina that was used.

### 2.2 TEM Imaging

**Figure 1:**
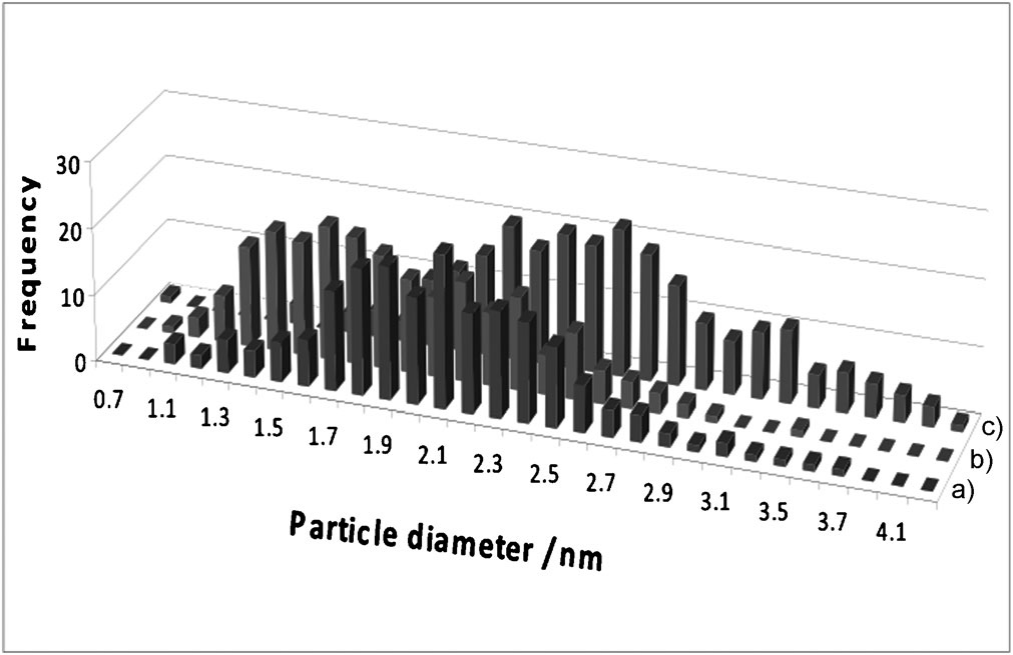
Particle-size distributions derived from TEM images for 4 wt % Rh catalysts: a) 4 wt % Rh/Al_2_O_3_, b) 4 wt % Rh/CeO_*x*_/Al_2_O_3_ (method I), and c) 4 wt % Rh/CeO_*x*_/Al_2_O_3_ (method II).

The TEM image for 4 wt % Rh supported on γ-alumina (Figure S2 in the Supporting Information) indicates that the supported rhodium catalyst consists of conglomerations of round platelets of alumina, which support, in a projection view, the presence of well-dispersed rhodium nanoparticles. The diameters of approximately 200 particles were measured per sample, with a minimum particle size of 0.5 nm (ca. 43 atoms). A collation of the particle size distributions, derived from the TEM images of 4 wt % Rh/Al_2_O_3_, 4 wt % Rh/CeO_*x*_/Al_2_O_3_ (method I), and 4 wt % Rh/CeO_*x*_/Al_2_O_3_ (mwthod II), is shown in Figure 1, and the relevant statistics of all of the rhodium systems are detailed in Table 1. The 4 wt % rhodium samples display relatively narrow particle-size distributions, with the size of the rhodium particles ranging from approximately 0.7 to 4 nm. The particle-size distributions for the 4 wt % Rh/Al_2_O_3_ and 4 wt % Rh/CeO_*x*_/Al_2_O_3_ (method I) samples display similar average metal particle sizes (ca. 2 nm); however, there is a clear shift towards a lower rhodium particle-size distribution for the ceriated rhodium catalyst (method I). However, the ceriated rhodium catalyst derived from method II shows a larger mean particle size (ca. 2.6 nm). Rhodium catalysts promoted with ceria and zirconia or just zirconia yield particle-size distributions between 1.5 and 3 nm, comparable to the non-promoted versions. The majority of the particles are around 2 nm in diameter.

**Table 1:** Rhodium particle-size distributions for the series of 4 wt % Rh samples, as determined by using TEM. Values in parenthesis are statistical errors.

Sample	No. of points	Mean particle size [nm]
4 wt % Rh/Al_2_O_3_	201	2.1(4)
4 wt % Rh/CeO_*x*_/Al_2_O_3_ (method I)	199	1.9(4)
4 wt % Rh/CeO_*x*_/Al_2_O_3_ (method II)	206	2.6(5)
4 wt % Rh/CeO_*x*_/ZrO_2_/Al_2_O_3_ (Ce/Zr=1:1)	201	2.1(3)
4 wt % Rh/CeO_*x*_/ZrO_2_/Al_2_O_3_ (Ce/Zr=2:1)	181	2.0(3)
4 wt % Rh/ZrO_2_/Al_2_O_3_	198	2.2(4)

A similar pattern is observed for 1.6 wt % Rh/γ-Al_2_O_3_ (Figure S3 in the Supporting Information), and distributions are shifted to lower sizes by approximately 0.2 nm for the zirconiated catalysts (Figure S4 in the Supporting Information). The particle sizes observed for the mixed-metal materials may represent a composite of the contributions from all components.

**Figure 2:**
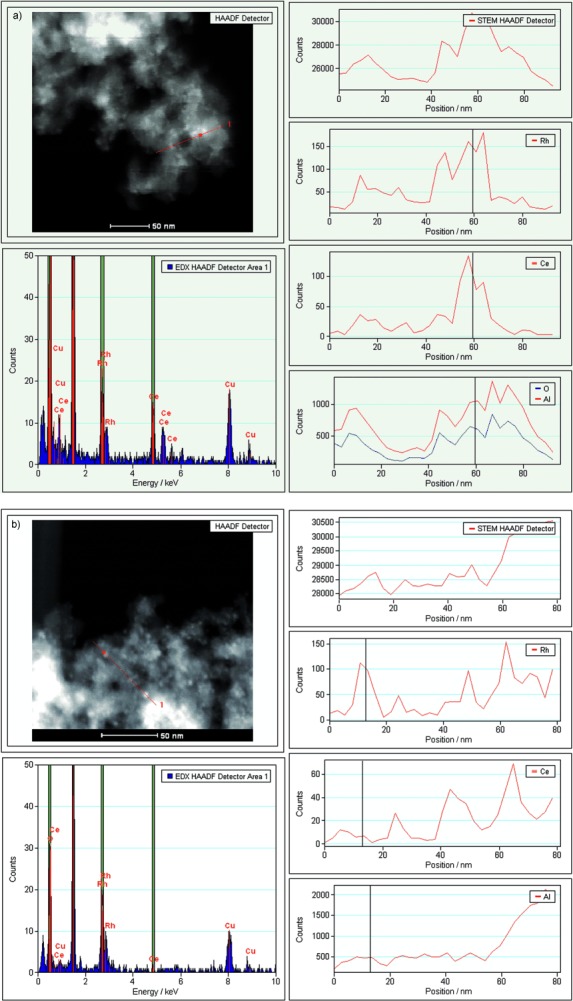
EDX and STEM–HAADF line profile analysis for a) 4 wt % Rh/CeO_*x*_/Al_2_O_3_ (method I) and b) 4 wt % Rh/CeO_*x*_/Al_2_O_3_ (method II). The upper left boxes show the STEM–HAADF images of the areas under investigation, and the EDX responses for the red lines are shown below the respective STEM–HAADF images. The EDX responses for each element across the red line are shown on the right-hand side.

Figure 2 a and b present STEM–HAADF and EDX line profile analyses for ceria-promoted catalysts with 4 wt % Rh/CeO_*x*_/Al_2_O_3_ prepared by method I and method II. For both catalysts, the EDX line profiles show that the Rh and Ce distributions are reasonably consistent with one another across the image: Ce peaks appear together with Rh; however, there are also isolated Rh peaks. This suggests that both these methods result in Rh centers that are in close proximity to the CeO_2_; however this intimate contact is not complete and some clusters are more indicative of the presence of Rh/γ-Al_2_O_3_. EDX maps for 1.6 wt % Rh/γ-Al_2_O_3_ (Figure S5 and S6 in the Supporting Information) support this hypothesis, showing the presence of Ce hotspots that are independent of the Rh signal, but in these cases there is an excess of Ce.

### 2.3 XPS and XANES

The electronic properties of the alumina-supported rhodium particles, as well as the effect of metal loading and ceria/zirconia doping, were studied by using XPS. The characteristic photoemission from the Ce 3d, Zr 3d, Rh 3d, O 1s, Cl 2p, Al 2p core levels were recorded for each sample. For consistency, all of the binding energies that are reported have been calibrated to the C 1s transition at 284.6 eV.^31^

#### Rh 3d Core-Level Spectra

**Figure 3:**
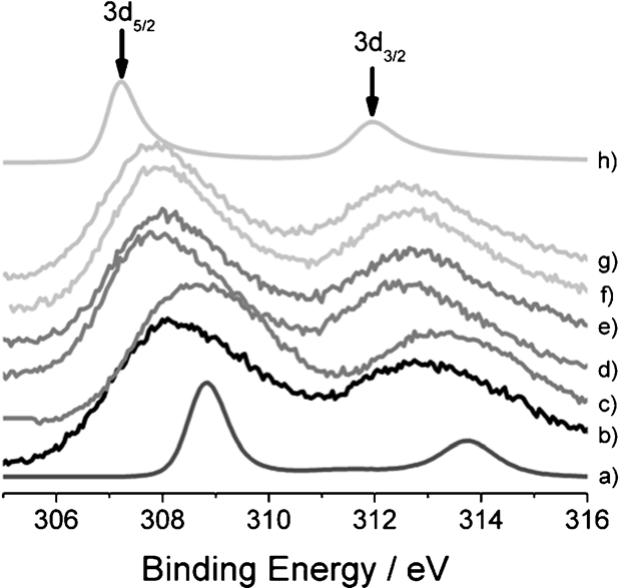
XPS spectra of Rh references and fresh 4 wt % Rh catalysts: a) Rh_2_O_3_, b) Rh/Al_2_O_3_, c) Rh/CeO_*x*_/Al_2_O_3_ (method I), d) Rh/CeO_*x*_/Al_2_O_3_ (method II), e) Rh/CeO_*x*_/ZrO_2_/Al_2_O_3_ (Ce/Zr=1:1), f) Rh/CeO_*x*_/ZrO_2_/Al_2_O_3_ (Ce/Zr=2:1), g) Rh/ZrO_2_/Al_2_O_3_, and h) Rh foil

The Rh 3d_5/2_ and 3d_3/2_ XPS spectra bands for Rh foil and Rh_2_O_3_ references, together with representative spectra for a series of fresh 4 wt % Rh catalysts, are shown in Figure 3. The results for the 1.6 wt % Rh samples are presented in Figure S7 in the Supporting Information. The positions of the two broad photoemission peaks indicate that, in all fresh Rh catalysts, there is a mixture of oxidation states.

All Rh 3d spectra have been fitted by using two sets of doublets that correspond to the metallic and oxidic Rh^3+^ chemical environments, with Rh 3d_5/2_ components centered around 307.5 and 308.9 eV binding energies, respectively, for 4 wt % Rh/γ-Al_2_O_3_ (Figure S8 in the Supporting Information). The energy differences between the 3d_3/2_ and 3d_5/2_ peaks were kept constant at the cited value of 4.5 eV. The full-width at half-maximum values (FWHMs) were 2.2±0.3 eV for the metallic component and 3.2 eV for oxidic Rh^3+^.^32^ After the curve fitting procedure, the fraction of Rh^3+^ for each rhodium catalyst was calculated by using the area ratio of the peaks derived from Rh^3+^ and the total area of the Rh 3d spectrum (Table 2). The calculated parameters indicate that Rh particles of the Rh/Al_2_O_3_ and Rh/CeO_*x*_/Al_2_O_3_ (method I) catalysts are extensively oxidized in air (ca. 80–90 %). However, there is a significantly larger fraction of metallic Rh present in the ceriated Rh catalysts produced by method II. This phenomenon is attributed to the synthesis method, wherein pre-reduced Rh particles supported on alumina are active for the Ce deposition through the surface modification method. This may relate to the synthesis procedure of method II, which does not involve calcination after the second high-temperature reduction step. The introduction of zirconia to the ceriated Rh catalysts does not cause any further reduction of Rh. However, the Rh particles in the Rh/ZrO_2_/γ-Al_2_O_3_ samples are the least oxidized among all of the Rh systems investigated.

**Table 2:** XPS and XANES oxidation-state fitting parameters for all Rh systems: the fraction of Rh^3+^ of the total of rhodium.

Sample	Rh^3+^/Rh^tot^ (XPS)	Rh^3+^/Rh^tot^ (XANES)
4 wt % Rh/Al_2_O_3_	0.78	0.82
4 wt % Rh/CeO_*x*_/Al_2_O_3_ (method I)	0.87	0.95
4 wt % Rh/CeO_*x*_/Al_2_O_3_ (method II)	0.56	0.54
4 wt % Rh/CeO_*x*_/ZrO_2_/Al_2_O_3_ (Ce/Zr=1:1)	0.56	0.46
4 wt % Rh/CeO_*x*_/ZrO_2_/Al_2_O_3_ (Ce/Zr=2:1)	0.62	0.52
4 wt % Rh/ZrO_2_/Al_2_O_3_	0.48	0.46

#### Rh K-Edge XANES

**Figure 4:**
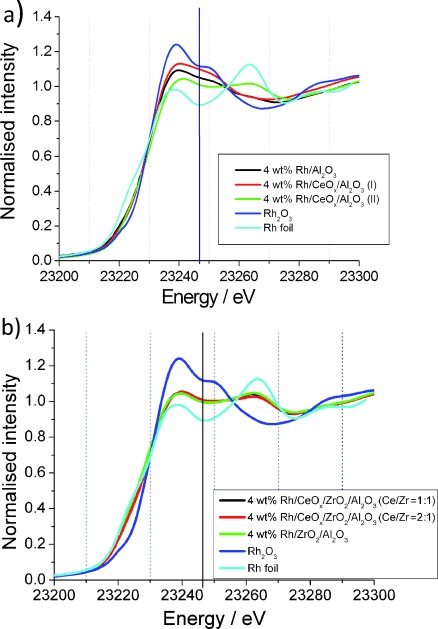
Rh K-edge XANES spectra for 4 wt % Rh catalysts: a) Rh/Al_2_O_3_, Rh/CeO_*x*_/Al_2_O_3_ (method I), and Rh/CeO_*x*_/Al_2_O_3_ (method II); b) Rh/CeO_*x*_/ZrO_2_/Al_2_O_3_ (Ce/Zr=1:1), Rh/CeO_*x*_/ZrO_2_/Al_2_O_3_ (Ce/Zr=2:1), and Rh/ZrO_2_/Al_2_O_3_. The spectra were acquired under ambient conditions. Rh_2_O_3_ and Rh foil are included for comparison.

Figure 4 a and b show the normalized XANES spectra obtained at the Rh K-edge for the Rh catalysts and the Rh reference materials. The energy of 23 246 eV was chosen for studying the XANES changes for each Rh catalyst. The XANES intensity of the Rh foil at this energy, representative for Rh^0^, was set to 0, and that of fully oxidized Rh_2_O_3_ was set to 1. As a result, the variation in XANES provided a scale for the degree of Rh oxidation. In agreement with the Rh XPS studies, the calculated XANES differences show (i) that the Rh/Al_2_O_3_ catalyst contains mostly oxidized Rh particles, (ii) the Rh particles of the ceriated Rh catalyst (method I) are the most oxidized, and (iii) in the Ce-doped Rh catalysts (method II) the Rh particles are the least oxidized. The XANES variations for Zr and Ce-promoted catalysts (Figure 4 b) show a similar trend, exhibiting Rh particles that are less oxidized. Moreover, the EXAFS edge positions of these samples are lower in energy, suggesting an overall lower Rh oxidation state compared to Rh/Al_2_O_3_ catalysts.

Table 2 shows the fraction of Rh^3+^ found in these systems on the basis of the XPS and XANES calculations. As indicated above, the results from both techniques are in agreement, indicating a similar level of Rh oxidation for the different Rh samples. However, for the Ce and Zr-doped Rh catalysts, XANES results showed slightly lower values for the Rh^3+^ fraction compared to the XPS data, which is beyond experimental error, and may be due to differing sample histories.

#### Ce 3d Core-Level Spectra

**Figure 5:**
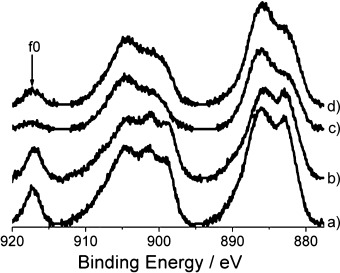
Ce 3d photoemission core-level spectra for ceriated 4 wt % Rh catalysts: a) Rh/CeO_*x*_/Al_2_O_3_ (method I), b) Rh/CeO_*x*_/Al_2_O_3_ (method II), c) Rh/CeO_*x*_/ZrO_2_/Al_2_O_3_ (Ce/Zr=1:1), d) Rh/CeO_*x*_/ZrO_2_/Al_2_O_3_ (Ce/Zr=2:1)

The Ce 3d XPS core-level spectrum is characteristic as it exhibits a three-lobed envelope at around 879–890 eV, 895–910 eV, and 917 eV, such as those depicted in Figure 5 for the 4 wt % Rh catalysts (the spectra of the 1.6 wt % Rh samples are presented in Figure S9 in the Supporting Information). The complex shape of each spectrum suggests that the Ce 3d spectrum arises from Ce with mixed +3 and +4 oxidation states.^33^, ^34^ The peak at the binding energy of 917 eV can be attributed to an initial state of the tetravalent Ce (called the f0 configuration) and it is, therefore, possible to differentiate between the two oxidation states. When comparing the intensity of the peak at around 917 eV for the range of Rh catalysts, it can be seen that for the ceria and zirconia-promoted samples versus the ceria-only doped catalysts, the peak is very small, indicating that the proportion of Ce^4+^ to that of Ce^3+^ is low. As previously reported, the incorporation of ZrO_2_ into a CeO_2_ lattice strongly promotes the reducibility of a mixed oxide and the oxygen mobility in the bulk.^35^, ^36^

**Figure 6:**
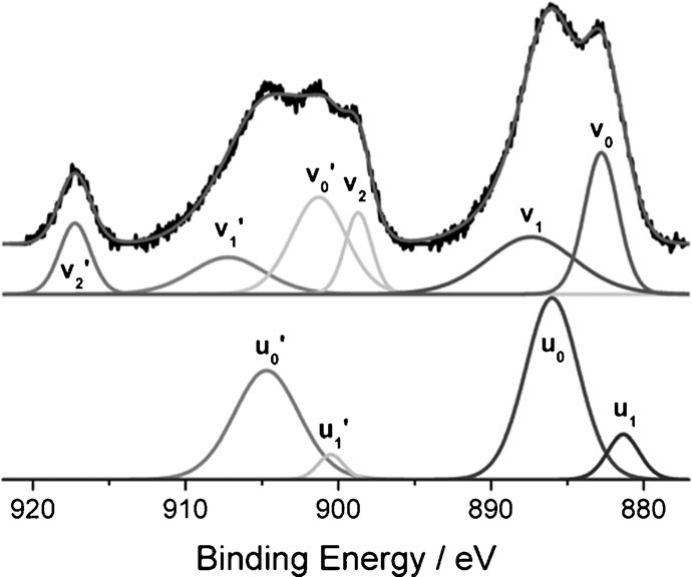
Fitted Ce 3d spectra of a representative Rh catalyst, Rh/CeO_x_/Al_2_O_3_ (method I), with the individual peak contributions corresponding to Ce^3+^ (u-lines) and Ce^4+^ (v-lines)

To quantify the two possible oxidation states of the Ce ions, it was necessary to curve-fit the Ce 3d_5/2_ and the Ce 3d_3/2_ spin-orbit doublet spectrum for each rhodium catalyst. The spectra were individually resolved and the features were grouped as v- (six peaks) and u- (four peaks) lines to depict the electronic transitions in Ce^4+^ and Ce^3+^, respectively (Figure 6). For Ce^4+^, the *v*_0_ and *v*_2_ components represent the intense peaks of the Ce 3d_5/2_ spin-orbit doublet, with a satellite, *v*_1_. Correspondingly, the *v*_0_′ and *v*_2_′ components characterize the Ce 3d_3/2_ doublet, and *v*_1_′ is the associated satellite. For a valence state of +3, the main components characterize the Ce 3d_5/2_ contribution, that is, *u*_0_ and the associated shake-down peak, *u*_1_. Furthermore, the Ce 3d_3/2_ doublet is indicated by the main peak, *u*_0_′, and its associated shake-down peak, *u*_1_′. The deconvolution of the Ce 3d spectrum was performed for each ceriated Rh catalyst and the peak positions, FWHMs, and relative contributions were derived from fitting the reference spectra of CeO_2_ for Ce^4+^ and Ce(acac)_3_ for Ce^3+^.

The relative amount of Ce^3+^ in the ceria supports and catalysts could be calculated by using an area ratio of the sum of the various peaks derived from Ce^3+^ to the total area of the Ce 3d spectrum. The calculation of a particular Ce oxidation state, estimated from the XPS data, is shown in Table 3. It can be observed that the ceria–alumina support, formed by using Ce(acac)_3_ as the precursor, mainly contains Ce^4+^ (ca. 70 %). Insertion of Zr into the CeO_2_ lattice decreases the amount of Ce^4+^ (ca. 40 %), irrespective of the amount of Zr that is inserted. Rhodium catalysts promoted by ceria form almost equivalent amounts of Ce^3+^ and Ce^4+^. During the deposition of 4 wt % Rh onto a ceria-alumina support (method I), the cerium seems to be partially reduced, resulting in a similar Ce^3+^/Ce^4+^ ratio to the sample prepared by using method II (ceria on Rh/Al_2_O_3_). Additional zirconia doping promotes the reducibility of ceria, and the dominant form in the system becomes Ce^3+^. The results obtained suggest that Ce and Zr are mostly present in this system in the form of a mixed oxide.

**Table 3:** Percentage area of the individual contributions for Ce^3+^ and Ce^4+^ in the total Ce 3d region.

Sample	Ce^3+^ [%]	Ce^4+^ [%]
support:		
5 wt % CeO_*x*_/Al_2_O_3_	27	73
5 wt % (Ce/Zr)/Al_2_O_3_ (Ce/Zr=1:1) (method II)	58	42
5 wt % (Ce/Zr)/Al_2_O_3_ (Ce/Zr=2:1) (method II)	60	40
catalyst:		
4 wt % Rh/CeO_*x*_/Al_2_O_3_ (method I)	47	53
4 wt % Rh/CeO_*x*_/Al_2_O_3_ (method II)	47	53
4 wt % Rh/CeO_*x*_/ZrO_2_/Al_2_O_3_ (Ce/Zr=1:1) (method II)	71	29
4 wt % Rh/CeO_*x*_/ZrO_2_/Al_2_O_3_ (Ce/Zr=2:1) (method II)	59	41

#### Cl 2p Core-Level Spectra

**Figure 7:**
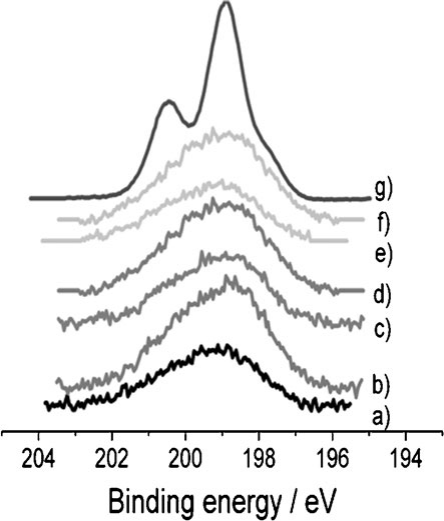
Cl 2p photoemission core-level spectra for 4 wt % Rh catalysts: a) Rh/Al_2_O_3_, b) Rh/CeO_*x*_/Al_2_O_3_ (method I), c) Rh/CeO_*x*_/Al_2_O_3_ (method II), d) Rh/CeO_*x*_/ZrO_2_/Al_2_O_3_ (Ce/Zr=1:1), e) Rh/CeO_*x*_/ZrO_2_/Al_2_O_3_ (Ce/Zr=2:1), f) Rh/ZrO_2_/Al_2_O_3_, together with g) RhCl_3_⋅3 H_2_O as a reference

The Cl 2p photoemission spectra for all of the Rh catalysts are shown in Figure 7, together with the Cl 2p spectrum of RhCl_3_.3H_2_O as a reference. The Cl 2p XPS spectrum of the RhCl_3_.3H_2_O presents two peaks derived from the Cl 2p_3/2_ and Cl 2p_1/2_ core-level regions centered at 198.0 and 200.6 eV, respectively. However, the spin-orbit coupling in the Cl 2p XPS spectra for the Rh catalysts is more difficult to see, and the bands are broader and of lower intensity. The center of the broad Cl 2p peak is found to be at a binding energy in the range of 199.1–199.7 eV, which correlates with previous literature;^37^ this broadening is likely to be associated with the distribution of sites. Among all of the Rh samples studied, the largest amount of Cl was observed for the ceriated Rh catalysts produced through method I (Figure 7, b), perhaps because of association within the ceria.

#### Zr 3d Core-Level Spectra

The Zr 3d XPS spectra for all of the Rh catalysts that have been promoted by zirconia, exhibit a spin-orbit doublet of the 3d level, which is split into 3d_5/2_ and 3d_3/2_ (Figure S10 and Table S2 in the Supporting Information). The Zr 3d_5/2_ peak is found to be centered within the binding energy range of 182.0–182.4 eV for all samples, which closely corresponds with the literature value of 182.2 eV for ZrO_2_.^38^ An extensive broadening is observed for the CeO_*x*_/ZrO_2_/γ-Al_2_O_3_ samples; this might be caused by strong interactions between zirconia and ceria, and/or with the rhodium particles. It may also be a result of static disorder owing to the poor crystallinity of the Zr components.

### 2.4 EXAFS Measurements

**Figure 8:**
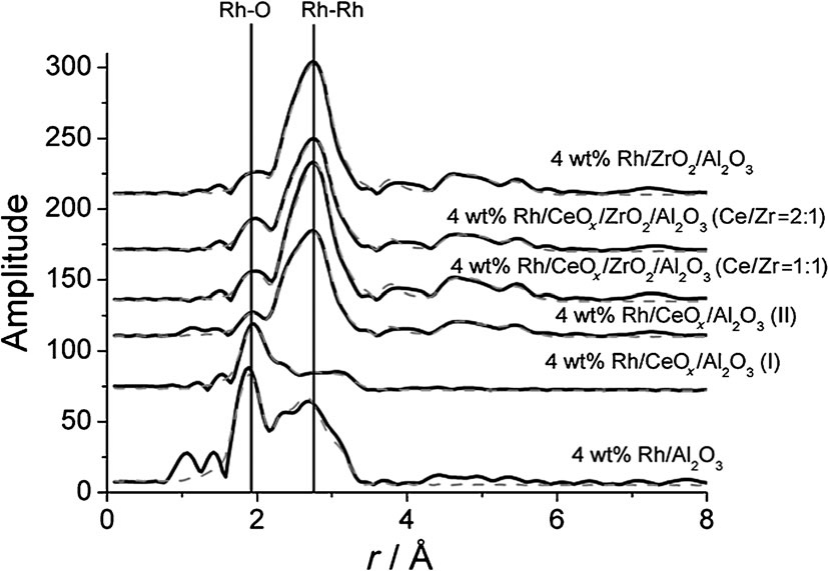
*k*^3^-weighted Rh K-edge Fourier transforms for the series of fresh (4 wt %) supported Rh catalysts (i.e. after calcinations, reduction, and exposure to air at RT). The data fitting, derived from analysis in EXCURV98, are shown by the dashed lines.

A detailed structural characterization of the Rh catalysts was performed by using Rh K-edge EXAFS measurements, which were performed on the Rh samples under ambient conditions (i.e. fresh samples) and following an in situ reduction at room temperature under H_2_ (i.e. reduced samples). The EXAFS results were used to determine the local structure of Rh atoms, as well as to identify the effects of ceria and zirconia. It is well known that Rh particles supported on alumina are easily oxidized after exposure to air.^39^–^41^ Therefore, the Rh—Rh coordination numbers that were determined by using EXAFS were utilized to estimate the amount of metallic Rh in the system. The Rh—O occupations were used to assess the amount of Rh that was oxidized. The Fourier-transform Rh K-edge EXAFSs for the 4 wt %-supported Rh samples are presented in Figure 8. The *k*^3^-weighted EXAFS (where *k* is the photoelectron wave number) and the corresponding fitting and Fourier transform data of the 1.6 wt % samples are shown in Figures S11 and S12 in the Supporting Information. The EXAFS data analysis was performed by using EXCURV98,^42^ and the structural fitting parameters for Rh catalysts studied under ambient conditions are detailed in Table 4 (for the 1.6 wt % samples, see Table S3 in the Supporting Information).

**Table 4:** Structural and statistical data derived from the analysis of Rh K-edge EXAFS over a series of 4 wt % Rh samples after calcination, reduction, and subsequent exposure to air at RT. Data range used was 2.5–15 k. *R*-fitting range=1–6 Å. AFAC (amplitude factor)=1. The *R* factor (goodness of fit) is given after the stepwise addition of shells. Values in parenthesis are statistical errors generated in EXCURV98. CN is coordination number.

Sample	Scatterer	*R* [Å]	CN	DW	*R* [%]	Sample	Scatterer	*R* [Å]	CN	DW	*R* [%]
Rh/Al_2_O_3_	O	2.02(1)	3.1(2)	0.010	64	Rh/CeO_*x*_/ZrO_2_/Al_2_O_3_ (Ce/Zr=1:1)	O	2.02(1)	1.7(2)	0.010	97
	Rh	2.67(1)	1.7(1)	0.011	32		Rh	2.68(1)	5.3(1)	0.011	25
	Cl	2.30(2)	0.4(1)	0.009	30		Rh	3.79(1)	1.3(2)	0.012	24
	Rh	3.06(1)	0.7(1)	0.012	26		Rh	4.66(1)	4.6(4)	0.012	17
							Rh	5.28(1)	4.7(5)	0.012	13
											
Rh/CeO_*x*_/Al_2_O_3_ (method I)	O	2.02(1)	3.6(2)	0.010	44	Rh/CeO_*x*_/ZrO_2_/Al_2_O_3_ (Ce/Zr=2:1)	O	2.03(1)	1.9(1)	0.010	94
	Rh	2.66(2)	1.0(1)	0.011	33		Rh	2.68(1)	4.4(1)	0.011	24
	Cl	2.33(1)	0.7(1)	0.009	27		Rh	3.79(1)	0.8(2)	0.012	22
	Rh	3.06(2)	0.7(1)	0.012	21		Rh	4.67(1)	3.5(4)	0.012	17
							Rh	5.28(1)	3.2(5)	0.012	13
											
Rh/CeO_*x*_/Al_2_O_3_ (method II)	O	2.02(1)	2.8(1)	0.010	84	Rh/ZrO_2_/Al_2_O_3_	O	2.03(1)	1.4(1)	0.010	97
	Rh	2.68(1)	3.2(1)	0.011	29		Rh	2.68(1)	5.1(1)	0.011	25
	Rh	3.80(2)	0.6(1)	0.012	27		Rh	3.78(1)	1.1(2)	0.012	22
	Rh	4.67(1)	2.3(5)	0.012	24		Rh	4.66(1)	4.1(4)	0.012	17
	Rh	5.28(1)	2.1(7)	0.012	22		Rh	5.27(1)	4.3(5)	0.012	14

Generally, the model that fits the experimental data for each Rh system contains two main shells, that is, a Rh—O shell at 2.02 Å and a Rh—Rh shell at 2.68 Å. The predominant Rh—O contribution at a distance of 2.02 Å indicates the presence of oxidic rhodium. The mean Rh—O bond length in the normal (hexagonal) form of Rh_2_O_3_ is. 2.04 Å,^43^ whereas in RhO_2_ there are two different Rh—O distances (four at 1.93 Å and two at 2.02 Å),^44^ so the results derived in this study are closer to Rh^III^. Other forms of Rh_2_O_3_ display considerable variation in reported Rh—O distances: 1.82–2.28 Å for a high-temperature form,^45^ and 1.99–2.10 Å for a high-pressure phase;^46^ however, in conjunction with the XPS results, the choice of Rh^III^ as the oxidized components is appropriate.

Moreover, we also observed a small Rh—Cl contribution at 2.35 Å for undoped Rh/Al_2_O_3_ and for the ceriated Rh catalyst (method I). A comparison of the fit, both with and without this shell, for a sample of 1.6 wt % Rh/γ-Al_2_O_3_ is presented in Figure S13 in the Supporting Information.

The presence of further Rh—Rh shells can be also determined for all Ce- and/or Zr-promoted Rh catalysts (method II), indicating the predominance of metallic Rh—Rh interactions for these materials with relatively large particle sizes. The observed Rh—Rh distances of 2.68, 3.79, 4.66, and 5.28 Å indicate the first four metal–metal distances in a fcc structure. These shells are only apparent for the materials containing zirconium for the 1.6 wt % series (Table S3). All of the other samples, with lower Rh loading, exhibit a Rh—Rh shell distance close to 3.06 Å. This shell was also evident in Rh/Al_2_O_3_ and Rh/CeO_*x*_/Al_2_O_3_ (method I) with 4 wt % Rh. A comparison of the Fourier transform (*k*^3^-weighted) EXAFS for the 1.6 wt % Rh/γ-Al_2_O_3_ and rhodium oxide phases is presented in Figure S14 in the Supporting Information. The supported rhodium does not possess the order observed for these phases extending beyond 3 Å. The ambient form of Rh_2_O_3_ contains two relatively close Rh—Rh distances at 2.72 and 2.99 Å (ratio 1:3),^43^ whereas RhO_2_ has Rh—Rh separations of 3.09 Å.^44^ Both the high-temperature and high-pressure forms of Rh_2_O_3_ show Rh—Rh separations of approximately 3.03 Å,^45^, ^46^ and our observed distance is in the expected region for a Rh—O—Rh interaction.

Exposure of Rh single crystals to oxygen has been shown to create a RhO_2_-like trilayer on the (111)^5^ and (110)^6^ surface planes. This structure exhibits longer range order than the supported rhodium catalysts (Figure S14), but it has been reported to display an expanded Rh⋅⋅⋅Rh distance of up to 3.10 Å, which can be attributed to the Rh—O—Rh bridge. Similar separations (3.02 Å) have been observed on stepped surfaces,^47^ and the structural pattern has also been modeled as a termination of the Rh_2_O_3_ (0001) plane.^48^ Such fractured structures are in agreement with the data on our supported catalysts.

**Figure 9:**
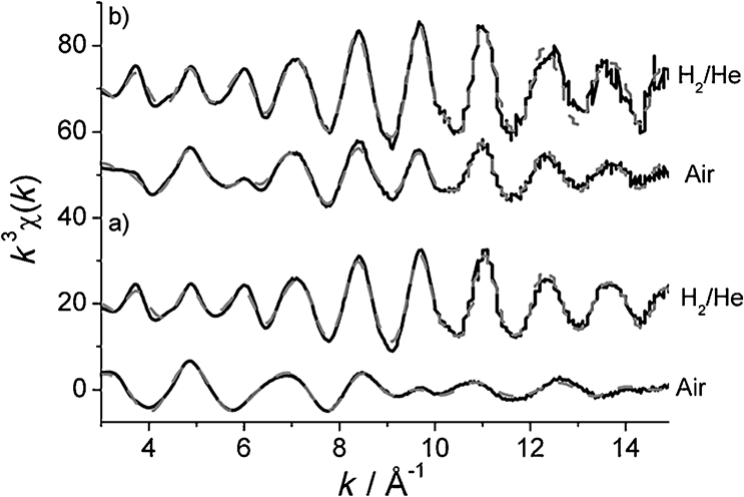
*k*^3^-weighted Rh K-edge EXAFS spectra for a) 4 wt % Rh/CeO_*x*_/Al_2_O_3_ (method I) and b) 4 wt % Rh/CeO_*x*_/Al_2_O_3_ (method II) in their fresh (air) and reduced states (maintained under 5 % H_2_/He at 295 K).

**Figure 10:**
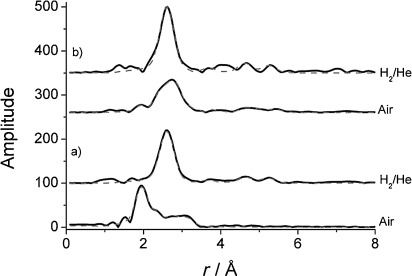
Fourier transforms of Rh K-edge EXAFS for a) 4 wt % Rh/CeO_*x*_/Al_2_O_3_ (method I) and b) 4 wt % Rh/CeO_*x*_/Al_2_O_3_ (method II) in their fresh (air) and reduced states (maintained under 5 % H_2_/He at 295 K).

The local coordination of Rh in the catalysts was also studied in a H_2_ atmosphere after reduction, and by using in situ Rh K-edge X-ray absorption spectroscopy, the coordination could be compared to their fresh analogues. The *k*^3^-weighted EXAFS data and Fourier transforms of the experimental data, with their corresponding theoretical fits, are reported in Figures 9 and 10, respectively. The corresponding EXAFS fitting parameters are shown in Table 5. The results for the 1.6 wt % series are given in Figures S15 and S16, as well as Table S4, in the Supporting Information. The EXAFS and Fourier transform signatures indicate that the local structure of Rh changes significantly under the two different conditions: (i) after air exposure (fresh) and (ii) after reduction in 5 % H_2_/He flow. The structural variations are especially evident for the undoped Rh/Al_2_O_3_ as well as the ceriated Rh catalyst (method I); the fresh samples resemble more oxidic rhodium, whereas an in situ-reduced sample displays a metallic structure. After in situ reduction in H_2_/He, the first shell Rh—Rh coordination number increases and no more oxygen is observed, indicating the formation of Rh metal particles. Moreover, the Rh—Rh shell at 3.06 A disappears and Rh—Rh distances become indicative of the fcc structure. For the 1.6 % series, the Rh—Rh shell near 3.06 Å was, again, not observed, and with the exception of 1.6 wt % Rh/CeO_v_/Al_2_O_3_ (method I), the same three non-bonded Rh—Rh shells could be included in the fit.

**Table 5:** Structural and statistical data derived from the analysis of the Rh K-edge EXAFS of a series of 4 wt % Rh samples under 5 % H_2_/He at RT. Data range used was 2.5–15 k. *R*-fitting range=1–6 Å. AFAC=1. Values in parenthesis are statistical errors generated in EXCURV98.

Sample	Scatterer	*R* [Å]	CN	*R* [%]	Sample	Scatterer	*R* [Å]	CN	*R* [%]
Rh/Al_2_O_3_	Rh	2.67(1)	6.8(2)	30	Rh/CeO_*x*_/ZrO_2_/Al_2_O_3_ (Ce/Zr=1:1)	Rh	2.68(1)	8.4(2)	23
	Rh	3.77(1)	1.7(2)			Rh	3.79(1)	1.8(1)	
	Rh	4.64(1)	4.7(1)			Rh	4.66(1)	5.6(2)	
	Rh	5.24(1)	3.6(1)			Rh	5.28(1)	5.8(2)	
									
Rh/CeO_*x*_/Al_2_O_3_ (method I)	Rh	2.67(1)	5.6(1)	24	Rh/CeO_*x*_/ZrO_2_/Al_2_O_3_ (Ce/Zr=2:1)	Rh	2.68(1)	7.4(2)	28
	Rh	3.77(1)	0.9(1)			Rh	3.77(1)	2.2(1)	
	Rh	4.65(1)	2.9(1)			Rh	4.65(1)	4.6(1)	
	Rh	5.27(1)	3.3(1)			Rh	5.26(1)	5.0(1)	
									
Rh/CeO_*x*_/Al_2_O_3_ (method II)	Rh	2.68(1)	6.9(2)	34	Rh/ZrO_2_/Al_2_O_3_	Rh	2.68(1)	7.9(2)	22
	Rh	3.78(1)	1.4(3)			Rh	3.79(1)	2.2(1)	
	Rh	4.66(2)	3.7(1)			Rh	4.66(1)	4.6(1)	
	Rh	5.29(3)	4.1(1)			Rh	5.28(1)	4.8(2)	

The average Rh—Rh coordination number for the first shell increases for all samples. An increase in coordination number from 1 to 5.6 can be observed for the 4 wt % Rh/CeO_*x*_/Al_2_O_3_ (method I) catalyst, and an increase from 2.8 to 6.9 occurs for the 4 wt % Rh/CeO_*x*_/Al_2_O_3_ (method II) catalyst. Furthermore, an increased Rh—Rh coordination number of 6.8 can be observed for Rh/Al_2_O_3_. In general, a similar trend is observed for all Rh catalysts; however, for each sample, different Rh—Rh coordination numbers are observed for the different shells, suggesting that different metal-particle sizes are obtained. The largest Rh particles are observed for the zirconiated and ceriated/zirconiated Rh catalysts, as the coordination number of first Rh—Rh shell is approximately 7–8, with the coordination numbers of their subsequent shells being significantly higher; this is also seen for the other samples. By using Jentys approach for estimating spherical particle sizes from coordination numbers,^49^ which is considered to be a good approximation,^50^ the particle sizes corresponding to the first coordination numbers were shown to relate to approximately 30–100 and 25–32 atoms for the 4 and 1.6 wt % Rh samples, respectively. This excludes the method I approach, which results in the lowest particle size. In all cases the coordination numbers for the second and third shells are lower than anticipated for a spherical growth pattern, implying lower order over >3 Å, and/or a more two-dimensional particle shape.

## 3. Discussion

The TEM measurements for the Rh/γ-Al_2_O_3_ catalysts show that the metal particles of each Rh catalyst have a similar, uniform size distribution in the range of 0.7–4 nm. The XPS, XANES, and EXAFS results for the fresh samples indicate that such particles supported on Al_2_O_3_ are rapidly oxidized, as previously discussed by Newton et al.^51^ A facile oxidation of Rh-supported samples, after exposure to air at room temperature, has previously been reported by using both energy dispersive^51^ and scanning EXAFS data.^9^, ^10^ The oxidized nature of Rh particles has implications in the interpretation of the TEM particle-size distributions for all Rh materials; the TEM images obtained do not discriminate between the metal and the oxide. Therefore, the measured pore-size distribution is a convolution of the relative amounts of oxide and metal in the system. These phases have significantly different volumes, thus obtaining an accurate assessment of the real atomicity of the Rh particles in each of these cases is complicated. This, however, has previously been recognized, and has been studied for Rh/Al_2_O_3_ catalysts.^52^ The XPS and XANES analyses indicate that the Rh particles obtained for the ceriated Rh catalyst (method I) are more extensively oxidized (ca. 90 % oxidation) than the particles for the undoped rhodium on alumina (ca. 80 % oxidation). However, the addition of promoters such as ceria and zirconia, introduced to the system by using method II (Ce and/or Zr deposition on pre-supported Rh on Al_2_O_3_), protect the Rh particles against extensive oxidation in air, forming mostly metallic Rh. The Rh—O bond lengths for the oxidic portion of the rhodium favor the Rh^III^ oxidation state, in agreement with the XPS measurements. The related Rh—Rh distance (3.06 Å), which represents the degree of local order of the oxidic region is reminiscent of oxidized sites on Rh single crystals.^5^, ^6^, ^47^, ^48^

The oxidic Rh structure of all of the fresh samples is easily reduced in an atmosphere of H_2_/He, and EXAFS studies confirm the presence of the fcc nanoparticle structure. In addition, the particle size is demonstrated to be a function of both the support and the preparation method.

XPS measurements show that some Cl is retained in the Rh systems of the fresh samples after the initial calcination and reduction processes; this is mostly evident for Rh/CeO_*x*_/Al_2_O_3_ (method I). The detailed EXAFS analysis shows a residual contribution of a Rh—Cl shell for two Rh catalysts: undoped Rh/Al_2_O_3_ and Rh/CeO_*x*_/Al_2_O_3_ (method I). The Rh—Cl contribution is not seen for the ceriated/zirconiated Rh samples that were prepared by using method II.

The EXAFS analyses show that Rh particles of the fresh Rh catalysts, excluding the undoped Rh/Al_2_O_3_ and the Rh/CeO_*x*_/Al_2_O_3_ (method I), can be simulated well by using a fcc structure, but with less local order than modeled by a spherical structure. For the 1.6 wt % series, with the exception of the ceriated sample, the average first Rh—Rh coordination numbers indicate a particle containing approximately 30 atoms. More variation for the particle size (30–110 atoms) is evident for the 4 wt % series, with the presence of zirconium favoring the larger particles.

Based on the EXAFS analysis, and previous findings,^9^, ^10^ it can be proposed that the supported Rh particles, after exposure to air at room temperature, are comprised of a metallic core surrounded by a thin oxide layer. Subsequent oxidation may only proceed by migration of dissociated oxygen through the oxide layer, and the metallic core is, therefore, only subject to relatively slow oxidation. This indicates that the addition of Ce, Zr, or Ce/Zr, by using method II, efficiently reduces the susceptibility of the Rh phase towards oxidation in air. The decomposition procedure (method II) yields this effect as a result of forming promoter oxides after the Rh nanoparticles have been synthesized on the γ-Al_2_O_3_. Hence, this may result in a partial covering of the reduced Rh nanoparticles by the promoter oxide phase, and, as a result, reduces its susceptibility to subsequent oxidation.

Method II materials are reducible through H_2_/He under mild conditions, and the rhodium remains accessible. However, EDX profile analysis performed for two ceriated Rh catalysts, prepared by method I and II, do not show any large discrepancies between the two systems, confirming that Rh and Ce sites are, generally, in close proximity to each other, which could suggest the presence of Rh—Ce redox couple. It has previously been found that the location of ceria close to Rh particles promotes Rh reduction by creating a surface oxygen vacancy.^53^

It has been demonstrated that Zr doping in Rh catalysts, which is always in the system in the +4 oxidation state, enhances the reducibility of CeO_*x*_. Quantitative analysis of the Ce 3d XPS spectra has shown that the fraction of Ce^3+^ increases from around 50 % for ceriated Rh catalysts to 60 % and 70 % for ceria–zirconia-promoted Rh catalysts with Ce/Zr ratios of 2:1 and 1:1, respectively. This phenomenon can be attributed to the structural perturbation of the CeO_2_ lattice by Zr incorporation, which enhances the oxygen mobility and forces Reaction (1):


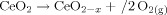
(1)

The redox properties of CeO_2_ in the presence of Zr have been also studied by Yang et al. through first-principles density functional theory.^36^ The Zr dopant was found to have significant effects on the ceria structure, and a substantial lowering of the oxygen-vacancy formation energy has been observed when the vacancy is created next to the Zr dopant. According to other quantum mechanical calculations,^54^ the most favorable location of Ce^3+^ is in the vicinity of the oxygen vacancies, which creates a driving force for the diffusion of oxygen. As previously investigated,^14^ the oxygen storage capability of cerium oxide plays a crucial role in enhancing the catalytic activity under reducing conditions. Therefore, the observed reducing effects of Zr on Ce, and the reducing effect of Ce and/or Zr on Rh particles, could explain the enhanced catalytic performance of the investigated Rh catalysts. It is the Rh/MO_*x*_(Ce/Zr=1:1)/Al_2_O_3_ sample that maintains both Ce and Rh in the lowest mean oxidation state.

## 4. Conclusions

The effects of the addition of ceria and/or zirconia on the structural properties of supported rhodium catalysts (1.6 and 4 wt % Rh/γ-Al_2_O_3_) were studied. The structures of the prepared catalyst materials were characterized ex situ by using N_2_ physisorption, TEM, STEM–HAADF, EDX, XPS, and EXAFS. All supported rhodium systems were demonstrated to readily oxidize in air at room temperature. By using ceriated and zirconiated precursors, a larger rhodium-based metallic core fraction was obtained, in comparison to the undoped rhodium catalysts, suggesting that ceria and zirconia could protect the rhodium particles against extensive oxidation. XPS results indicated that after the calcination and reduction treatments, a small amount of chlorine was retained on the support of all rhodium catalysts, and EXAFS analysis showed significant Rh—Cl interactions for Rh/Al_2_O_3_ and Rh/CeO_*x*_/Al_2_O_3_ (method I) catalysts. After reaction with H_2_/He in situ, for series of samples with 1.6 wt % Rh, EXAFS first shell analysis indicated a mean size of approximately 30 atoms. A broader spread was evident with a 4 wt % Rh loading (ca. 30–110 atoms), with the incorporation of zirconium providing the largest particle sizes.

## Experimental Section

### Sample Preparation

**Rh/γ-Al_2_O_3_:** 4 wt % supported Rh/γ-Al_2_O_3_ samples were prepared through wet impregnation of Al_2_O_3_ (Degussa, Alumina C, surface area ca. 88 m^2^ g^−1^; 1.92 g) with RhCl_3_⋅3 H_2_O (0.21 g) in aqueous solution. The solution was stirred by using a Teflon-coated magnetic stirrer until a uniform paste was achieved. The sample was then dried in air. Subsequently, the resultant mixture was calcined for 6 h at 673 K in 5 % O_2_/He, and then reduced for 5 h under flowing 5 % H_2_/He at 573 K.

**Ceriated Rh/γ-Al_2_O_3_ (method I):** 5 wt % Ce/γ-Al_2_O_3_ support was produced by dissolving cerium(III)2,4-pentanedionate (0.509 g) in toluene and γ-Al_2_O_3_ (1.805 g) was then added. The sample was dried overnight in the air before being calcined under 5 % O_2_/He for 6 h at 500 °C. Subsequently, the solution of RhCl_3_⋅H_2_O in water was added to a suspension of the Al_2_O_3_/CeO_2_ support, and this mixture was then stirred. The sample was dried overnight in air before being calcined under 5 % O_2_/He for 6 h at 773 K, and then it was reduced under 5 % H_2_/He for 5 h at 300 °C.

**Ceriated/zirconiated Rh/γ-Al_2_O_3_ (method II):** Rh/γ-Al_2_O_3_ ( 1 g) was re-reduced under flowing 5 % H_2_/He for 3 h at 573 K. To prepare 5 wt % Ce-containing catalysts, a solution of Ce(acac)_3_ (0.164 g) in toluene (100 mL) was placed in a three-way tap dropper, which was purged by N_2_ for 15 min, and this was then added drop-wise to the reduced catalyst. Subsequently, the reagents were mixed under flowing 5 % H_2_/He at 353 K for 8 h. Then, the sample was filtered and dried in air overnight. The sample was again reduced under 5 % H_2_/He at 573 K for 3 h. All preparations were performed under a N_2_ atmosphere. In order to produce Rh catalysts doped by Zr, Zr(acac)_4_ (0.281 g) was dissolved in toluene (100 mL). Catalysts promoted by ceria and zirconia of two different mixture ratios (Ce/Zr=1:1 and 2:1) were produced by simultaneously dissolving the appropriate amount of Ce(acac)_3_ and Zr(acac)_4_ in toluene.

All calcined/reduced Rh samples that were exposed to air are denoted in this paper as fresh samples, and these were then characterized by using BET surface area measurements, TEM, XPS, and EXAFS techniques. All of the subsequent samples were reduced in situ (in a DRIFTS cell) for the EXAFS measurements. These samples are denoted reduced. The in situ reduction was performed as follows: reduction occurred in 5 % H_2_/He at 573 K followed by oxidation under 5 % O_2_/He at 573 K until the remaining carbonaceous deposits were removed from the catalyst (performed by observing carbon-related fragments in the mass spectra), after which the flow was changed back to 5 % H_2_/He before cooling to room temperature.

**BET surface area measurements:** The surface area data was obtained by using a Micrometrics GEMINI III 2375 surface-area analyzer.

**TEM:** An electron microscope (Tecnai F20) at the Johnson Matthey Technology Centre was used to obtain the TEM images. The microscope was operated at 200 keV with a point resolution of <2 Å. It was also equipped with a HAADF detector for STEM, as well as an EDX detector. This instrumentation is capable of detecting even very small metal particles (ca. 3 nm) through Z contrast and by analyzing selected points by using EDX spectroscopy. The fresh supported Rh samples were dispersed in ethanol and deposited on a perforated carbon foil, which was supported on a copper grid.

**XPS:** The Scienta ESCA300 Spectrometer at NCESS in Daresbury Laboratory, UK, was used to acquire the XPS data. The instrument used a high-power rotating anode and a monochromatised AlK_α_ (*hν*=1486.7 eV) X-ray source with energy resolution of 0.35 eV. Samples were used in the powder form (on adhesive tape), and mounted onto a stub. The stub was loaded into the XPS system through a load-lock, which was evacuated before transfer to the analysis chamber. The analysis chamber was maintained at a base pressure of approximately 5×10^−9^ mbar. The atomic percentages derived from XPS were compared with those from EDX, and have been provided in Table S5 in the Supporting Information.

**X-ray absorption spectroscopy:** Rh K-edge EXAFS spectra were mainly measured in transmission at the European Synchrotron Radiation Facility (ESRF) in Grenoble, France at BM29, as well as at beamline 9.3 of the Synchrotron Radiation Source (SRS) in Daresbury, UK, and at B18 of Diamond Light Source, UK. The measurements were performed in transmission mode by using optimized ionization chambers as the detectors.

**Data handling and analysis:** Data reduction was performed by using Xmult,^55^ and analysis was carried out through a spherical wave formalism by using EXCURV98.^39^ The *R*-factors quoted are defined as *R*=(∫[*χT*−*χE*]*kndk*/[*χE*]*kndk*)⋅100 %, in which *χT* and *χE* are the theoretical and experimental EXAFS, k is the photoelectron wave vector, *dk* is the range of photoelectron wave vectors analyzed, and *n* is the weighting in *k* that was applied to the data. The number of parameters, *N*, that can be justifiably fit was estimated from the Nyquist equation: *N*=(2Δ*k*Δ*r*/π)+1, in which Δ*k* and Δ*r* are the ranges in *k*- and *r*-space over which the data are analyzed. DW factors for Rh—Rh, Rh—O, and Rh—Cl shells were estimated for Rh/Al_2_O_3_, and subsequently the spectra for the whole range of Rh catalysts were analyzed in the same *k* range by holding the DW factor constant (2 *σ*^2^=0.012 Å^2^).

The proportion of Rh^III^ and Rh metal, as shown in Table 2, was estimated from Rh K-edge XANES by using the relative absorption at 23.31 keV.^56^
