# Supplementary material for: Structural Characterization of Alumina-Supported Rh Catalysts: Effects of Ceriation and Zirconiation by using Metal–Organic Precursors
Source: Chemphyschem. 2013 Aug 13;14(15):3606–17. doi: 10.1002/cphc.201300537 (PMC3935408; doi:10.1002/cphc.201300537)
Supplement: Supplementary file 1 [file cphc0014-3606-sd1.pdf]

CHEMPHYSCHEM

Supporting Information

© Copyright Wiley-VCH Verlag GmbH & Co. KGaA, 69451 Weinheim, 2013

Structural Characterization of Alumina-Supported Rh Catalysts: Effects of Ceriation and Zirconiation by using Metal–Organic Precursors

Anna B. Kroner,[a, d] Mark A. Newton,*[b] Moniek Tromp,[c, d] Andrea E. Russell,[d]
Andrew J. Dent,[a] and John Evans*[a, d, e]

cphc_201300537_sm_miscellaneous_information.pdf

Supporting Information

"Structural characterisation of alumina supported Rh catalysts: Effects of ceriation and zirconiation by metalorganic precursors"

Table S1. BET surface area results for the sample investigated

Sample	BET surface area [m^2/g]
γ-Al_2O_3	88
5 wt% Ce/γ-Al_2O_3	81
5 wt% Ce/Zr/γ-Al_2O_3	89
4 wt% Rh/Al_2O_3	95
4 wt% Rh/CeO_x/Al_2O_3 (method I)	70
4 wt% Rh/CeO_x/Al_2O_3 (method II)	89
1.6 wt% Rh/Al_2O_3	91
1.6 wt% Rh/CeO_x/Al_2O_3 (method I)	68
1.6 wt% Rh/CeO_x/Al_2O_3 (method II)	82

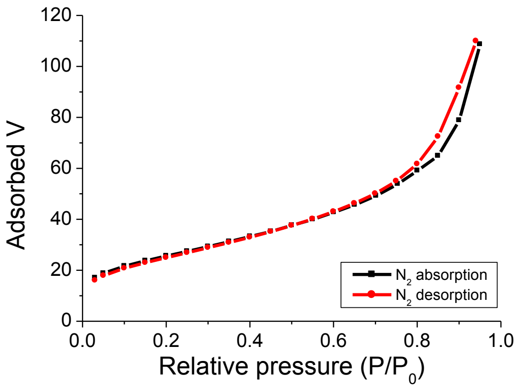

Figure S1. N_2 adsorption/desorption isotherm for 1.6 wt% Rh/Al_2O_3

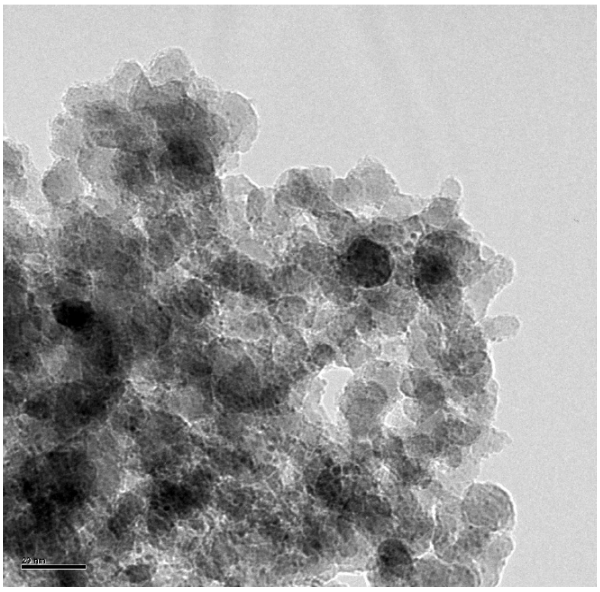

Figure S2. TEM image of 4 wt% Rh/γ-Al_2O_3. Scale bar: 2 nm.

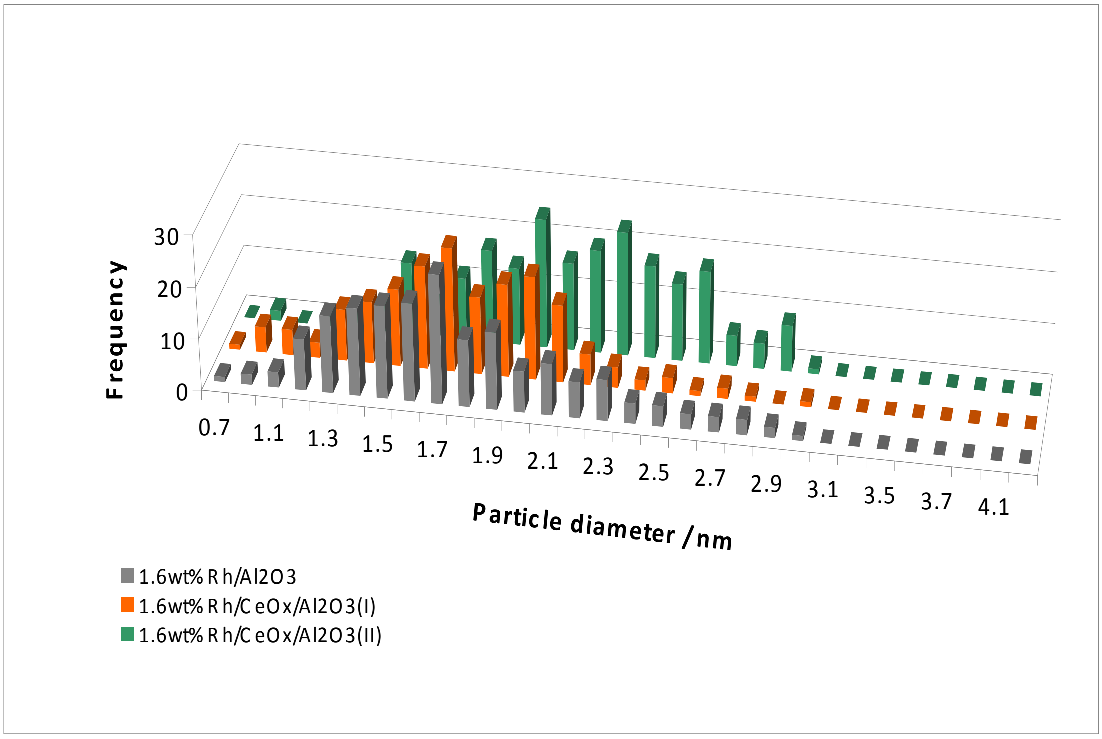

Figure S3. Particle size distributions derived from TEM for 1.6 wt% Rh catalysts

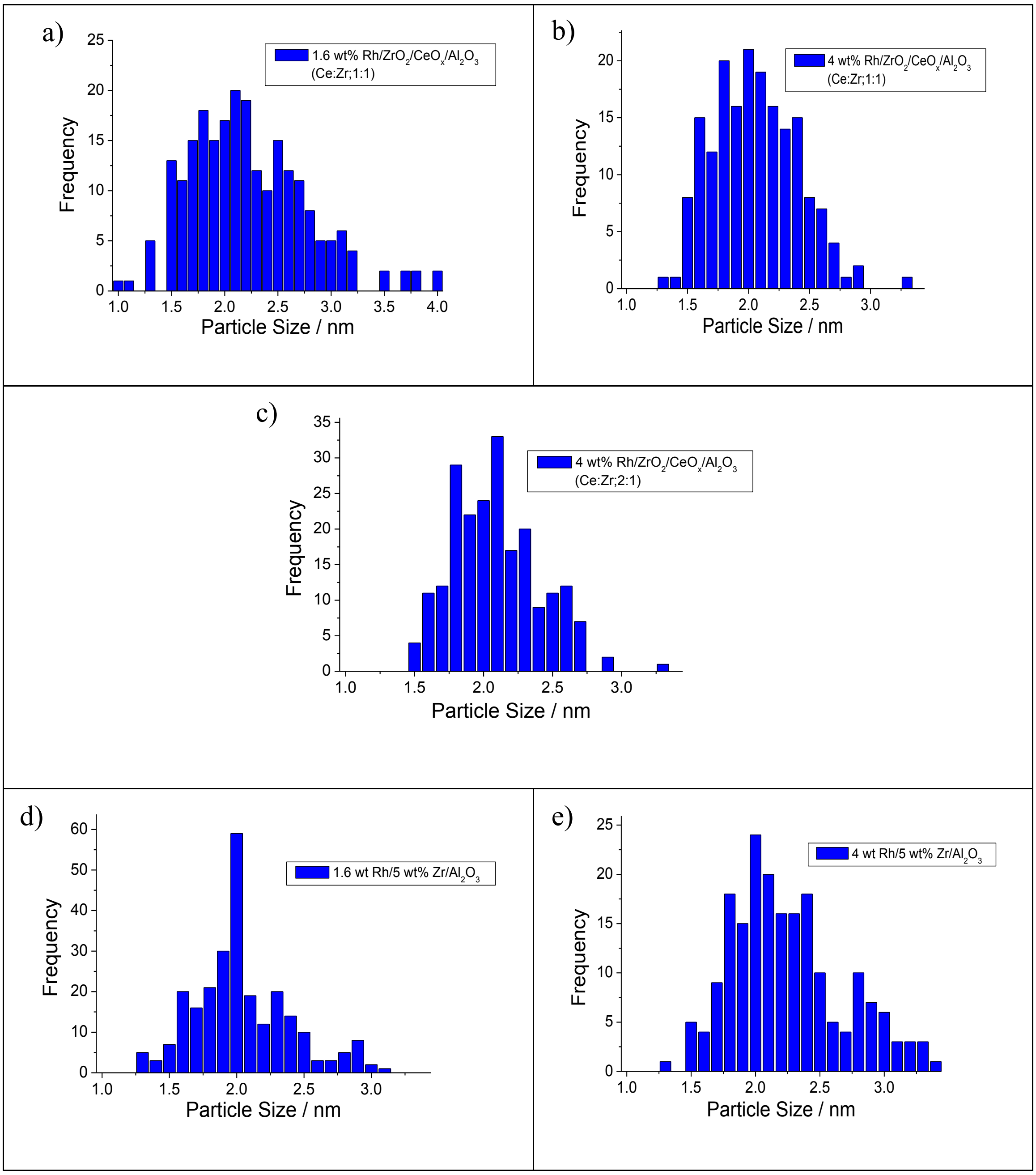

Figure S4. Particle size distributions of 1.6 and 4 wt% Rh catalysts doped by ceria and zirconia, or zirconia only.

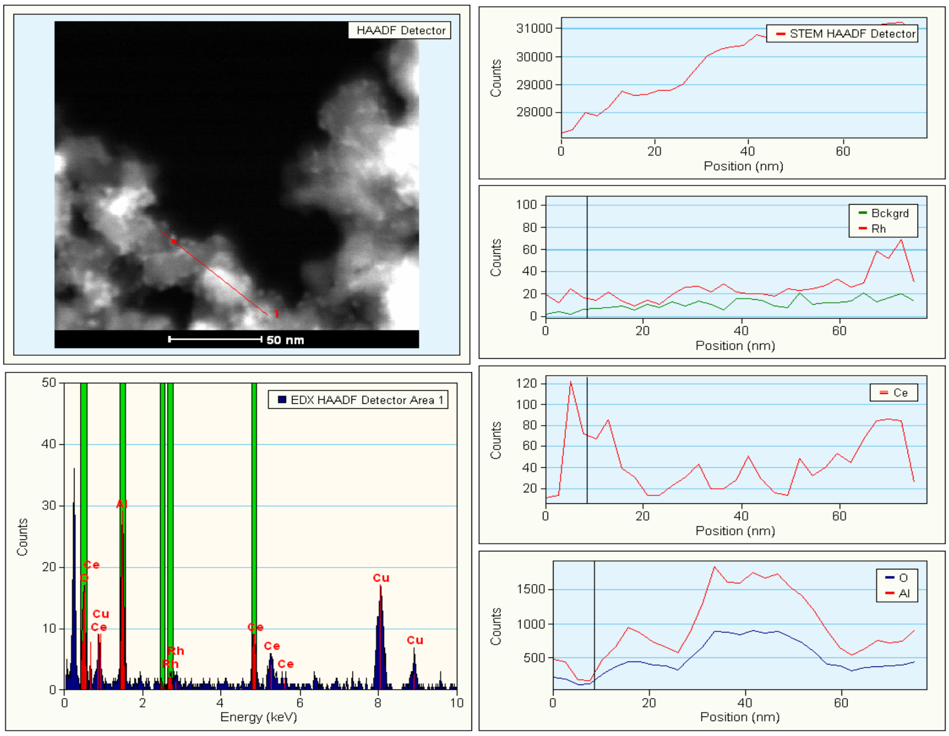

Figure S5. STEM EDX HAADF line profile analysis for 1.6 wt% Rh/CeO_x/Al_2O_3 (method I). The upper left box shows the STEM HAADF image of the area under investigation. The HAADF EDX response for the red line labelled 1 is shown below the STEM image. The HAADF EDX response across the red line are shown on the right hand side and in descending order show total counts, counts from Rh, Ce and the last plot indicate O and Al trend.

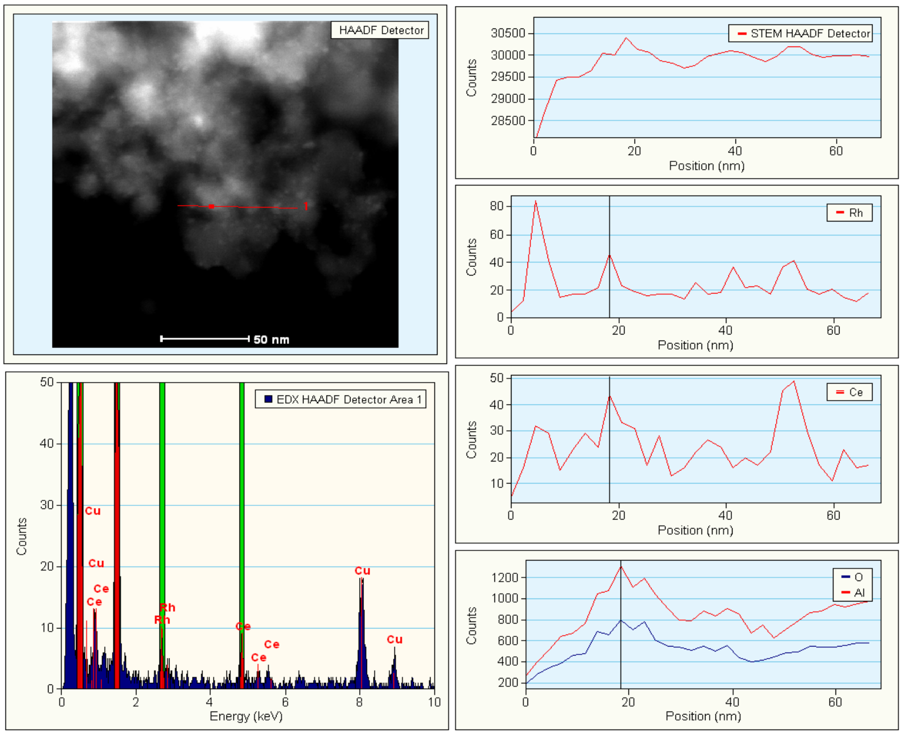

Figure S6. STEM EDX HAADF line profile analysis for 1.6 wt% Rh/CeO_x/Al_2O_3 (method II). The upper left box shows the STEM HAADF image of the area under investigation. The HAADF EDX response for the red line labelled 1 is shown below the STEM image. The HAADF EDX response across the red line are shown on the right hand side and in descending order show total counts, counts from Rh, Ce and the last plot indicate O and Al trend.

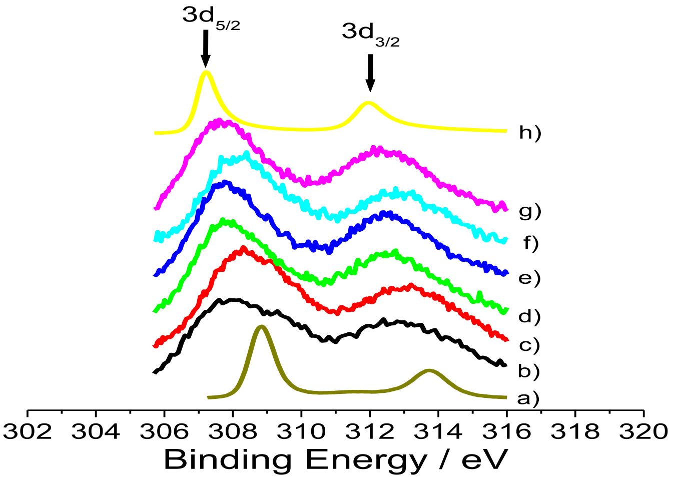

Figure S7. XPS spectra of the fresh catalysts: a) Rh_2O_3, b) 1.6 wt% Rh/Al_2O_3, c) 1.6 wt% $Rh/CeO_x/Al_2O_3$ (I), d) 1.6 wt% $Rh/CeO_x/Al_2O_3$ (II), e) 1.6 wt% $Rh/CeO_x/ZrO_2/Al_2O_3$ (Ce:Zr;1:1),
f) 1.6 wt% $Rh/CeO_x/ZrO_2/Al_2O_3$ (Ce:Zr;2:1), g) 1.6 wt% $Rh/ZrO_2/Al_2O_3$,h) Rh foil

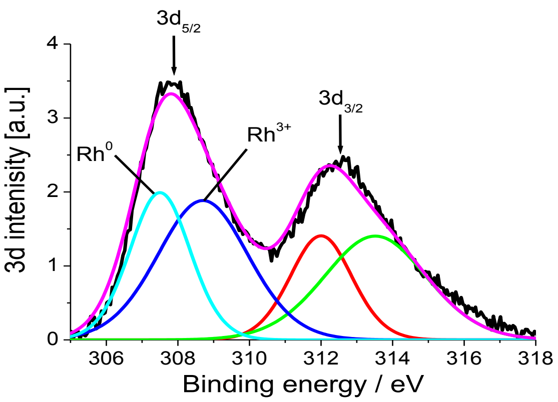

Figure S8. Fitted Rh 3d XPS spectra of 4 wt% Rh/γ-Al_2O_3.

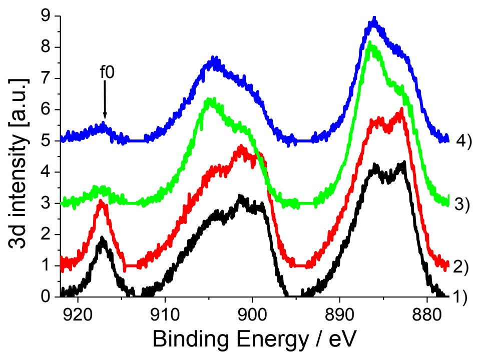

Figure S9. Ce 3d photoemission core level spectra for; a) 1.6 wt% Rh catalysts: 1) $Rh/CeO_x/Al_2O_3$ (I), 2) $Rh/CeO_x/Al_2O_3$ (II), 3) $Rh/CeO_x/ZrO_2/Al_2O_3$ (Ce:Zr;1:1), 4) $Rh/CeO_x/ZrO_2/Al_2O_3$ (Ce:Zr;2:1)

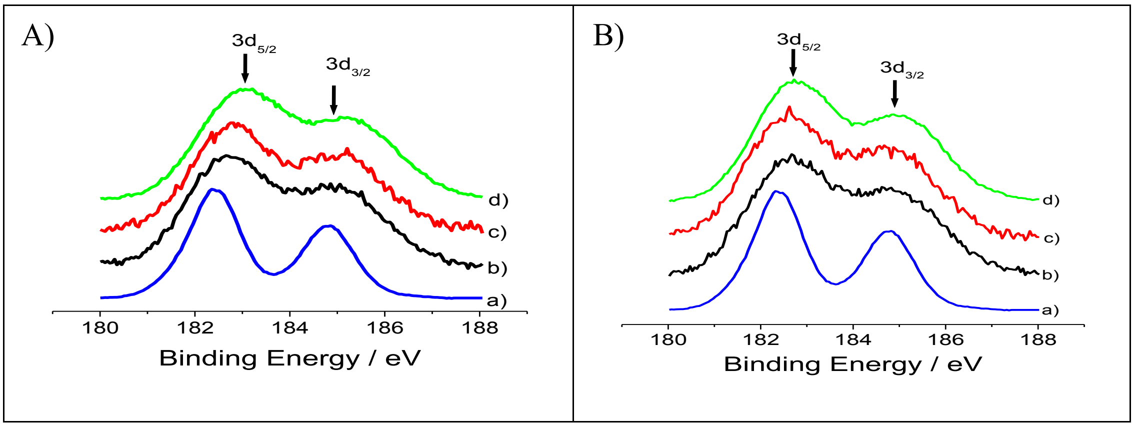

Figure S10. Zr 3d photoemission core level spectra for A) 1.6 wt% Rh catalysts B) 4 wt% Rh catalysts: a) ZrO_2, b) $Rh/CeO_x/ZrO_2/Al_2O_3$ (Ce:Zr;1:1), c) $Rh/CeO_x/ZrO_2/Al_2O_3$ (Ce:Zr;2:1), d) $Rh/ZrO_2/Al_2O_3$

Table S2. Energy positions of the spectral components in Zr $3d_{5/2}$ and Zr $3d_{3/2}$ observed in rhodium catalysts

Sample	Zr ($3d_{5/2}$) [eV]	Zr ($3d_{3/2}$) [eV]
$Zr(acac)_4$	182.2	184.6
5 wt% Zr/Al_2O_3	182.1	184.5
5 wt% $(Ce/Zr)/Al_2O_3$ (Ce:Zr; 1:1)	182.4	184.8
5 wt% $(Ce/Zr)/Al_2O_3$ (Ce:Zr; 2:1)	182.4	184.8
1.6 wt% $Rh/CeO_x/ZrO_2/Al_2O_3$ (Ce:Zr;1:1)	182.1	184.5
1.6 wt% $Rh/CeO_x/ZrO_2/Al_2O_3$ (Ce:Zr;2:1)	182.1	184.5
1.6 wt% $Rh/ZrO_2/Al_2O_3$	182.3	184.7
4 wt% $Rh/CeO_x/ZrO_2/Al_2O_3$ (Ce:Zr;1:1)	182.0	184.4
4 wt% $Rh/CeO_x/ZrO_2/Al_2O_3$ (Ce:Zr;2:1)	182.1	184.5
4 wt% $Rh/ZrO_2/Al_2O_3$	182.1	184.5

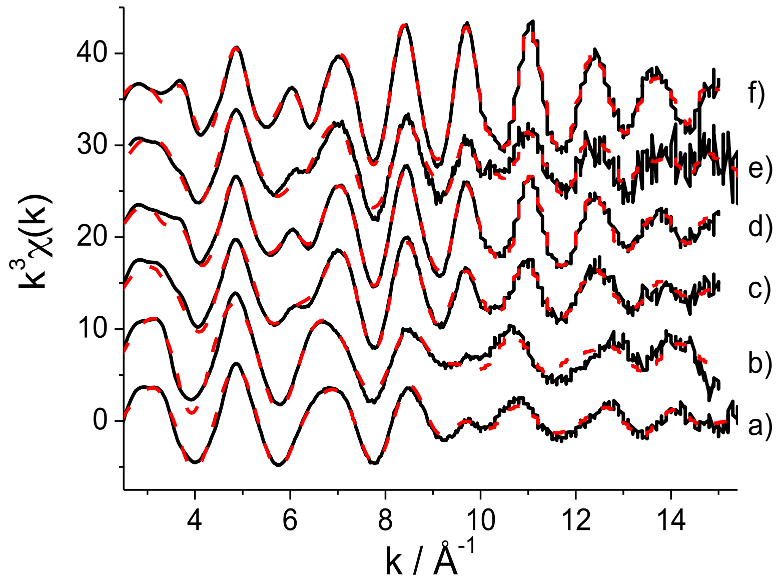

Figure S11. Rh K edge EXAFS derived from the range of 1.6 wt% Rh catalysts synthesised in this study after the calcinations and reduction procedures, at RT exposure to air. Fits to the data, derived from analysis in EXCURV are shown in red. a) 1.6 wt% Rh/Al_2O_3, b) 1.6 wt% $Rh/CeO_x/Al_2O_3$ (I), c) 1.6 wt% $Rh/CeO_x/Al_2O_3$ (II), d) 1.6 wt% $Rh/CeO_x/ZrO_2/Al_2O_3$ (Ce:Zr;1:1), e) 1.6 wt% $Rh/CeO_x/ZrO_2/Al_2O_3$ (Ce:Zr;2:1), f) 1.6 wt% $Rh/ZrO_2/Al_2O_3$

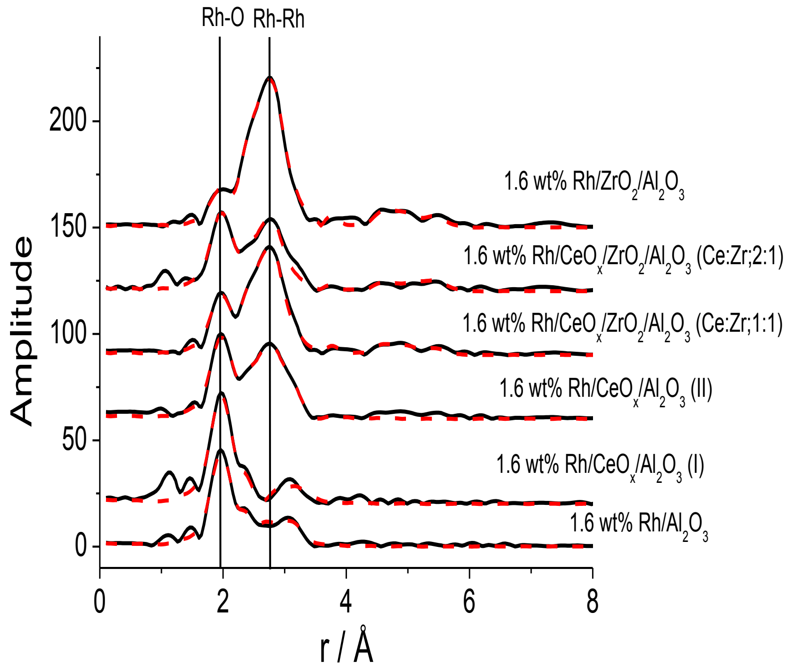

Figure S12. Fourier transforms of k^3 weighted Rh K edge EXAFS derived from the range of 1.6 wt% Rh catalysts synthesised in this study after the calcinations and reduction procedures, at RT exposure to air. Fits to the data, derived from analysis in EXCURV are shown in red.

Table S3. Structural and statistical data derived from the analysis of Rh K edge EXAFS over a series of 1.6 wt% Rh samples after calcination, reduction, and subsequent exposure to air at RT. Data range used was 2.5 – 13 k. R fitting range 1 – 6 Å. AFAC = 1. The R factor is given after the stepwise addition of shells. Values in parenthesis are statistical errors generated in EXCURV98.

Sample	Rh-E	R / Å	CN	DW	R / %	Sample	Rh-E	R / Å	CN	DW	R / %
**Rh/Al_2O_3**	O	2.02 (1)	3.6 (2)	0.010	47	**$Rh/CeO_x/ZrO_2$ $/Al_2O_3$ (Ce:Zr;1:1)**	O	2.03 (1)	2.4 (1)	0.010	85
	Rh	2.65 (1)	0.9 (2)	0.011	41		Rh	2.67 (1)	2.8 (1)	0.011	25
	Cl	2.31 (2)	0.6 (1)	0.009	30		Rh	3.77 (3)	0.3 (2)	0.012	25
	Rh	3.05 (1)	0.8 (1)	0.012	22		Rh	4.66 (1)	1.5 (4)	0.012	22
							Rh	5.27 (2)	1.5 (5)	0.012	21
**$Rh/CeO_x/$ Al_2O_3 (I)**	O	2.04 (1)	4.4 (2)	0.010	35	**$Rh/CeO_x/ZrO_2$ $/Al_2O_3$ (Ce:Zr;2:1)**	O	2.03 (1)	3.0 (2)	0.010	71
	Rh	2.63 (2)	0.4 (1)	0.011	33		Rh	2.66 (1)	1.8 (1)	0.011	34
	Cl	2.35 (1)	0.5 (1)	0.009	27		Rh	3.06 (1)	0.4 (2)	0.012	32
	Rh	3.06 (2)	0.8 (1)	0.012	21						
**$Rh/CeO_x/$ Al_2O_3 (II)**	O	2.03 (1)	3.2 (1)	0.010	69	**$Rh/ZrO_2/Al_2O_3$**	O	2.03 (1)	1.6 (1)	0.010	94
	Rh	2.66 (1)	2.0 (1)	0.011	26		Rh	2.67 (1)	3.9 (1)	0.011	23
	Rh	3.06 (1)	0.7 (1)	0.012	20		Rh	3.78 (1)	0.4 (2)	0.012	22
							Rh	4.65 (1)	2.2 (4)	0.012	20
							Rh	5.27 (1)	2.3 (6)	0.012	17

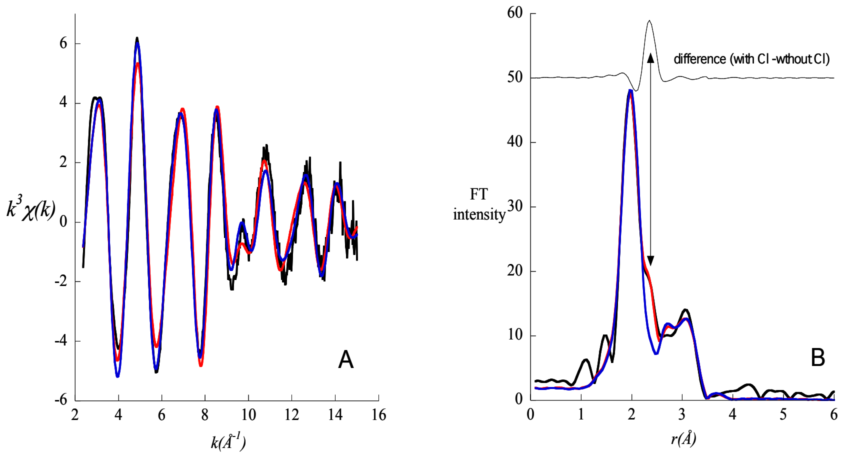

Figure S13. The rhodium K edge k^3-weighted EXAFS (A) and Fourier transform (B) for 1.6 wt% Rh/Al_2O_3. The experimental data is given by the black line. Two theoretical fits are shown: one with a Rh-Cl contribution (red) and one with no Rh-Cl component (blue). In panel (B) the difference between the FT's from each of these model is shown.

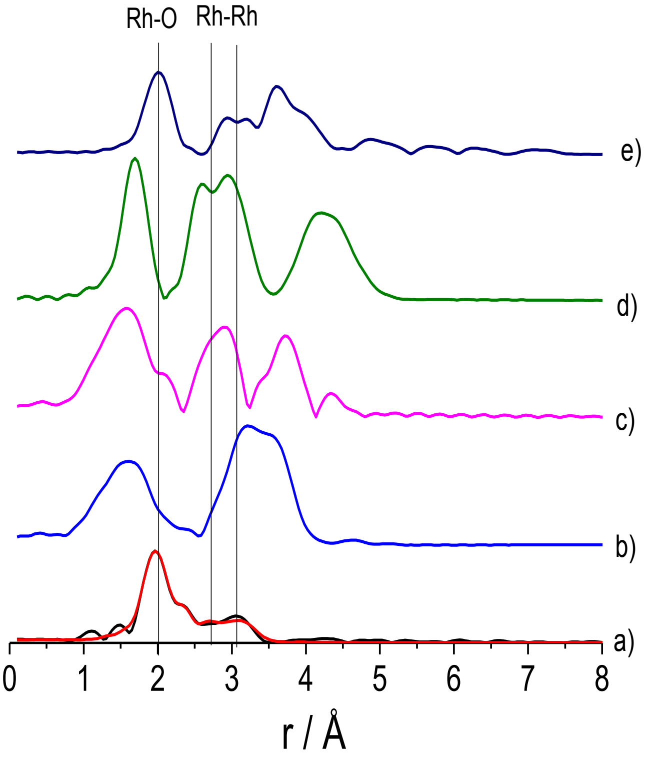

Figure S14. Fourier transform for a) 1.6 wt% Rh/Al_2O_3 together with the fit derived from analysis in EXCURV98 (Figure S13). FT spectra from four possible Rh oxides – as indicated – are shown sequentially above such as b) Rh_2O_3 ortho (Shannon), c) Rh_2O_3 (Coey), d) RhO_2 (Shannon), e) RhO_2 trilayer (Lundgren). The solid vertical lines highlight the correspondence between the experimental data and the known "bulk" oxides for first shell Rh-O, and first and second shell Rh-Rh contributions.

[References:

Lundgren: J. Gustafson, A. Mikkelsen, M. Borg, J. N. Andersen, E. Lundgren, C. Klein, W. Hofer, M. Schmid, P. Varga, L. Kohler, G. Kresse, N. Kasper, A. Stierle, H. Dosch, *Phys. Rev. Lett.*, **2005**, *71*, 115442,1-9.

RhO_2: R. D. Shannon, *Solid State Comm.*, **1968**, *30*, 121-125

Rh_2O_3 (HEX.): G. Lunde, *Z. Anorg. Allg. Chem.*, **1927**, *163*, 345-350.

Rh_2O_3 (Ortho Shannon): R. D. Shannon, C .T. Prewitt, *J. Solid State Chem.*, **1970**, 2**,** 134-136.

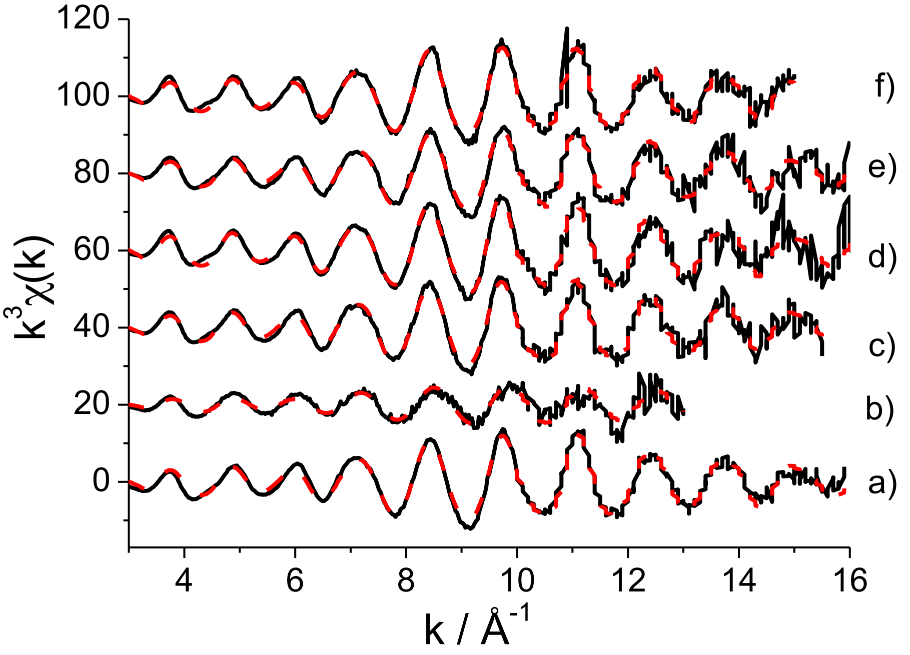

Figure S15. Rh K edge EXAFS derived from the range of 1,6 wt% Rh catalysts under 5 % H_2/He at RT. Fits to the data, derived from analysis in EXCURV are shown in red. a) 1,6 wt% Rh/Al_2O_3, b) 1,6 wt% Rh/CeO_x/Al_2O_3 (I), c) 1,6 wt% Rh/CeO_x/Al_2O_3 (II), d) 1,6 wt% Rh/CeO_x/ZrO_2/Al_2O_3 (Ce:Zr;1:1), e) 1,6 wt% Rh/CeO_x/ZrO_2/Al_2O_3 (Ce:Zr;2:1), f) 1,6 wt% Rh/ZrO_2/Al_2O_3

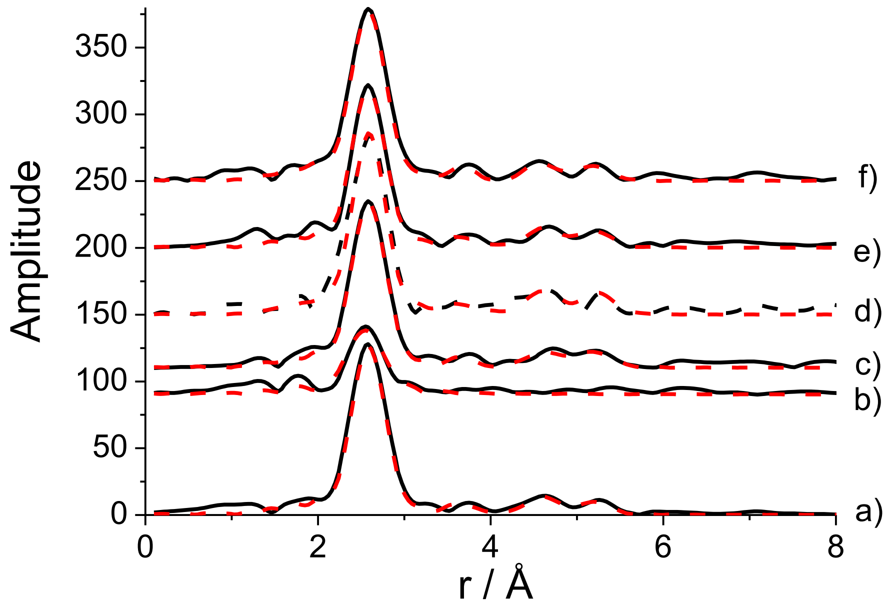

Figure S16. Fourier transform of the Rh K edge EXAFS derived from the range of 1,6 wt% Rh catalysts under 5 % H_2/He at RT. Fits to the data, derived from analysis in EXCURV are shown in red. a) 1,6 wt% Rh/Al_2O_3, b) 1,6 wt% Rh/CeO_x/Al_2O_3 (I), c) 1,6 wt% Rh/CeO_x/Al_2O_3 (II), d) 1,6 wt% Rh/CeO_x/ZrO_2/Al_2O_3 (Ce:Zr;1:1), e) 1,6 wt% Rh/CeO_x/ZrO_2/Al_2O_3 (Ce:Zr;2:1), f) 1,6 wt% Rh/ZrO_2/Al_2O_3

Table S4. Structural and statistical data derived from the analysis of Rh K edge EXAFS derived from a range of 1.6 wt% Rh samples under 5 % H_2/He at RT. Data range used was 2.5-15 k.

Sample	Rh-E	CN	R / A	R / %	Sample	Rh-E	CN	R / A	R / %
**1.6 wt% Rh/Al_2O_3**	Rh	6.4 (1)	2.66 (1)	21	**1.6 wt% Rh/CeO_x/ZrO_2/Al_2O_3 (Ce:Zr;1:1)**	Rh	6.7 (2)	2.66 (2)	15
	Rh	1.1 (2)	3.76 (1)			Rh	1.1 (1)	3.74 (2)	
	Rh	3.3 (3)	4.63 (1)			Rh	4.2 (2)	4.65 (2)	
	Rh	3.4 (2)	5.25 (1)			Rh	4.6 (2)	5.28 (2)	
**1.6 wt% Rh/CeO_x/Al_2O_3 (I)**	Rh	2.9 (2)	2.64 (2)	41	**1.6 wt% Rh/CeO_x/ZrO_2/Al_2O_3 (Ce:Zr;2:1)**	Rh	6.5 (2)	2.66 (1)	13
						Rh	1.3 (2)	3.78 (2)	
						Rh	3.5 (2)	4.65 (3)	
						Rh	4.4 (4)	5.24 (2)	
**1.6 wt% Rh/CeO_x/Al_2O_3 (II)**	Rh	6.9 (2)	2.67 (3)	22	**1.6 wt% Rh/ZrO_2/Al_2O_3**	Rh	6.6 (2)	2.66 (2)	14
	Rh	1.3 (1)	3.75 (2)			Rh	1.2 (1)	3.78 (2)	
	Rh	2.9 (3)	4.66 (2)			Rh	3.3 (1)	4.63 (1)	
	Rh	4.7 (2)	5.24 (1)			Rh	3.9 (2)	5.25 (1)	

Table S5. Analytical data derived from EDX and XPS techniques and compared with the initial composition.					
**Sample**	**Element**	**Wt % (EDX)**	**Atom % (EDX)**	**Atom % (XPS)**	**Atom % (initial)**
1.6 wt% Rh/Al_2O_3	Rh	2.0 (4)	0.4	0.5	0.3
	Cl	1.1 (2)	0.6	0.8	0.9
	Al	50.9 (4)	39.3	40.2	39.5
	O	46.2 (4)	59.6	58.5	59.2
4 wt% Rh/Al_2O_3	Rh	4.3 (5)	0.9	1	0.8
	Cl	0.5 (2)	0.5	0.8	2.4
	Al	49.6 (4)	38.8	40.2	38.7
	O	45.3 (4)	59.6	58	58
1.6 wt% $Rh/CeO_x/Al_2O_3$ (I)	Rh	2.0 (5)	0.4	0.5	0.3
	Cl	1.2 (2)	0.8	0.9	1
	Al	46.0 (5)	37.8	37.8	37.8
	Ce	8.6 (5)	1.4	1.5	1.3
	O	42.5 (5)	59.5	59.2	59.5
4 wt% $Rh/CeO_x/Al_2O_3$(I)	Rh	5.1 (5)	1.1	1.2	0.9
	Cl	1.6 (2)	1	1.7	2.6
	Al	44.1 (5)	37.2	36.9	36.9
	Ce	7.4 (5)	1.2	1.3	1.3
	O	41.7 (5)	59.7	58.8	58.3
1.6 wt% $Rh/CeO_x/Al_2O_3$ (II)	Rh	2.0 (5)	0.9	0.5	0.4
	Cl	0.8 (2)	0.5	0.4	0.9
	Al	47.5 (5)	38.4	41	39.3
	Ce	6.1 (5)	1	1	0.8
	O	43.7 (6)	59.7	57.1	58.6
4 wt% $Rh/CeO_x/Al_2O_3$(II)	Rh	4.1 (5)	0.9	1	0.9
	Cl	0.9 (2)	0.6	0.3	2.5
	Al	46.5 (5)	38.1	40.9	38.4
	Ce	5.2 (5)	0.8	1.1	0.8
	O	43.1 (5)	59.6	56.7	57.4
1.6 wt% $Rh/CeO_x/ZrO_2/Al_2O_3$ (Ce:Zr;1:1)	Rh	1.8 (4)	0.4	0.5	0.3
	Cl	0.6 (3)	0.4	0.3	1
	Al	48.9(8)	39.9	41.3	39.1
	Ce	2.5 (6)	0.6	0.7	0.4
	Zr	1.9 (8)	0.5	1	0.6
	O	44.3 (8)	58.2	56.2	55.5
4 wt% $Rh/CeO_x/ZrO_2/Al_2O_3$ (Ce:Zr;1:1)	Rh	4.3 (5)	0.9	1.1	0.8
	Cl	0.8 (2)	0.5	0.5	2.3
	Al	47.1 (5)	38.2	39.5	35.5
	Ce	2.2 (3)	0.3	0.7	0.4
	Zr	2.0 (4)	0.5	1	0.6
	O	43.6 (5)	59.6	57.6	53.1
1.6 wt% $Rh/CeO_x/ZrO_2/Al_2O_3$ (Ce:Zr;2:1)	Rh	1.6 (4)	0.4	0.4	0.3
	Cl	0.5 (2)	0.3	0.4	1
	Al	48.8 (7)	38.8	40.4	39.1
	Ce	3.4 (3)	0.7	0.7	0.5
	Zr	1.6 (4)	0.6	0.6	0.4
	O	44.1 (6)	59.2	57.5	58.6
4 wt% $Rh/CeO_x/ZrO_2/Al_2O_3$ (Ce:Zr;2:1)	Rh	4.0 (5)	0.9	1	0.9
	Cl	0.7 (2)	0.4	0.5	2.5
	Al	46.2 (6)	37.7	39.2	38.3
	Ce	3.3 (4)	0.5	0.8	0.5
	Zr	1.2 (3)	0.3	0.7	0.4
	O	43.7 (6)	60.2	57.8	57.3
1.6 wt% $Rh/ZrO_2/Al_2O_3$	Rh	3.4 (5)	0.7	1	0.9
	Cl	0.9 (2)	0.6	0.5	2.5
	Al	46.9 (6)	37.8	39.4	38.2
	Zr	4.6 (7)	1.1	1.9	1.2
	O	44.2 (5)	59.8	57.2	57.2
4 wt% $Rh/ZrO_2/Al_2O_3$	Rh	3.4 (5)	0.7	1	0.9
	Cl	0.9 (2)	0.6	0.5	2.5
	Al	46.9 (6)	37.8	39.4	38.2
	Zr	4.6 (7)	1.1	1.9	1.2
	O	44.2 (5)	59.8	57.2	57.2
